# Comparative physiological, metabolomic, and transcriptomic analyses reveal mechanisms of apple dwarfing rootstock root morphogenesis under nitrogen and/or phosphorus deficient conditions

**DOI:** 10.3389/fpls.2023.1120777

**Published:** 2023-06-19

**Authors:** Bin Xie, Yanhui Chen, Yanzhen Zhang, Xiuhong An, Xin Li, An Yang, Guodong Kang, Jiangtao Zhou, Cungang Cheng

**Affiliations:** ^1^ Key Laboratory of Mineral Nutrition and Efficient Fertilization for Deciduous Fruits, Liaoning Province/Key Laboratory of Fruit Germplasm Resources Utilization, Ministry of Agriculture and Rural Affairs/Research Institute of Pomology, Chinese Academy of Agricultural Sciences, Xingcheng, Liaoning, China; ^2^ Research Center for Agricultural Engineering Technology of Mountain District of Hebei/Mountainous Areas Research Institute, Hebei Agricultural University, Baoding, Hebei, China

**Keywords:** apple dwarfing rootstock, nitrogen and phosphorus deficiencies, root architecture, cell wall, expansin

## Abstract

Nitrogen (N) and phosphorus (P) are essential phytomacronutrients, and deficiencies in these two elements limit growth and yield in apple (*Malus domestica* Borkh.). The rootstock plays a key role in the nutrient uptake and environmental adaptation of apple. The objective of this study was to investigate the effects of N and/or P deficiency on hydroponically-grown dwarfing rootstock ‘M9-T337’ seedlings, particularly the roots, by performing an integrated physiological, transcriptomics-, and metabolomics-based analyses. Compared to N and P sufficiency, N and/or P deficiency inhibited aboveground growth, increased the partitioning of total N and total P in roots, enhanced the total number of tips, length, volume, and surface area of roots, and improved the root-to-shoot ratio. P and/or N deficiency inhibited ${\text{NO}}_{3}^{\;-}$ influx into roots, and H^+^ pumps played a important role in the response to P and/or N deficiency. Conjoint analysis of differentially expressed genes and differentially accumulated metabolites in roots revealed that N and/or P deficiency altered the biosynthesis of cell wall components such as cellulose, hemicellulose, lignin, and pectin. The expression of *MdEXPA4* and *MdEXLB1*, two cell wall expansin genes, were shown to be induced by N and/or P deficiency. Overexpression of *MdEXPA4* enhanced root development and improved tolerance to N and/or P deficiency in transgenic *Arabidopsis thaliana* plants. In addition, overexpression of *MdEXLB1* in transgenic *Solanum lycopersicum* seedlings increased the root surface area and promoted acquisition of N and P, thereby facilitating plant growth and adaptation to N and/or P deficiency. Collectively, these results provided a reference for improving root architecture in dwarfing rootstock and furthering our understanding of integration between N and P signaling pathways.

## 1 Introduction

Nitrogen (N) and phosphorus (P) are two major phytomacronutrients required for plant growth and development (Feng et al., 2022; Wang et al., 2022). Nitrate ( ${\text{NO}}_{3}^{\;-}$ ) is the main source of N in aerobic soils (Bouguyon et al., 2015; Lu et al., 2021), but its availability can fluctuate dramatically in both time and space (Lv et al., 2021). ${\text{NO}}_{3}^{\;-}$ deficiency inhibits photosynthesis, accelerates leaf senescence, alters root architecture, induces expression of ${\text{NO}}_{3}^{\;-}$ transporters *NRT1.1* and *NRT2* to promote N absorption capacity, and affects most enzyme activities required for energy metabolism (Wang et al., 2019a; Wang et al., 2020; Zhao et al., 2020; Nezamivand-Chegini et al., 2022; Wen et al., 2022). Phosphate (Pi), the major inorganic form of P taken up by roots, has low mobility due to its affinity for cations and its conversion to organic forms (Zhang et al., 2021d). Pi deficiency negatively affects structural compounds, delays leaf development, restricts plant growth, and changes root architecture (Epie et al., 2019; Sun et al., 2021b; Nezamivand-Chegini et al., 2022). Several genes, such as Pi transporter *PHT* and *SPX* family members, as well as transcription factors, such as P starvation response (*PHR*), are involved in the response to Pi deficiency (Sun et al., 2017b; Kumar et al., 2021; Xiao et al., 2021; Li et al., 2022; Liu et al., 2022).

${\text{NO}}_{3}^{\;-}$ and Pi are also important signaling molecules, and their signaling pathways interact at several levels (Hu et al., 2019; Krouk and Kiba, 2020; Torres-Rodriguez et al., 2021). Accumulating evidence suggests that the P starvation response (PSR) strongly depends on N availability (Hannam et al., 2018; Hu et al., 2019; Medici et al., 2019; Ueda et al., 2020). ${\text{NO}}_{3}^{\;-}$ inducible transcription factors, such as HRS1, Hox52, and GLK1, affect the PSR and root development, thereby regulating Pi uptake in response to P deficiency (Medici et al., 2015; Li et al., 2022; Wei et al., 2022). Medici et al. (2015) reported that AtNIGT1, a GRAP transcription factor, can be regulated by both ${\text{NO}}_{3}^{\;-}$ and Pi. AtNIGT1 was able to regulate the expression of ${\text{NO}}_{3}^{\;-}$ response genes and Pi-starvation-inducible genes (PSIs), and coordinate the utilization of N and P. In addition, AtNIGT1 together with its close homolog (HHO1) involved in modulating root development by regulating the expression of downstream genes. Therefore, through the module integrating ${\text{NO}}_{3}^{\;-}$ and Pi, which constructed with AtNIGT1 as the center, plant can simultaneously sense the changes in N and P availability and regulate root development by comprehensive commands.

Root is the first organ that perceives and takes up nutrients in plants (Nasr Esfahani et al., 2021). Plant root cell wall is a solid matrix wall structure that is made up of protein and polysaccharide biopolymers, and it is the first barrier to resist biotic and abiotic stress (Yan et al., 2022). When roots come into contact with the soil, root cell walls alter their composition, as the ion exchange groups in the cell wall polymers react with ions in the soil (Ogden et al., 2018; Meychik et al., 2021). A lack of nutrient elements such as N, P, boron, calcium, iron, etc. impairs cell wall formation and cell growth (Fang et al., 2019; Qin et al., 2019; Zhu et al., 2019; Liu et al., 2021b; Rivai et al., 2021; Chen et al., 2022a; Li et al., 2023; Nezamivand-Chegini et al., 2023). The integrity of plant cell wall also affected the root formation process (Devi et al., 2021). Expansins are cell-wall-loosening proteins, they interact with the primary cell wall, which is composed of cellulose, hemicellulose, pectin, and xyloglucan. Expansins can promote the non-covalent interactions between cellulose microfibrils, which weaken and move against each other, leading to loosening of the tight cellulosic structure (Chen et al., 2022b). Previous reports have provided evidences that expansins were not only associated with environmental stress tolerance in plants such as nutrient deficiency, drought, salt, etc., but involved in root development (Han et al., 2014; Kong et al., 2019; Ding et al., 2021; Liu et al., 2021a; Wu et al., 2021; Tian et al., 2022). Although previous studies have provided insights into the physiological and molecular processes of apple in response to ${\text{NO}}_{3}^{\;-}$ or Pi deficiency (Valentinuzzi et al., 2019; Sun et al., 2021b; Zhang et al., 2021b; Tahir et al., 2022; Wen et al., 2022), the exact metabolite profiles in root remain unknown. In agricultural systems, plants often suffer from N and Pi co-limitation. Combined N and P deficiencies lead to a series of adaptive responses that cannot be ascribed simply to the combined deficiency (Pueyo et al., 2021; Nasr Esfahani et al., 2022). In chickpea, although molecular responses under combined N and P deficiency are weaker than those seen under deficiency of a single nutrient, simultaneous deficiency produces unique metabolic characteristics (Nasr Esfahani et al., 2021; Nasr Esfahani et al., 2022). Investigations of transcriptome responses to combined N and P deficiency have been performed in *Arabidopsis thaliana* (Medici et al., 2015), rice (*Oryza sativa*) (Cai et al., 2013), *Medicago truncatula* (Bonneau et al., 2013), and giant duckweed (*Spirodela polyrhiza*) (Yang et al., 2022), etc. The structural and functional modifications of roots that occur under combined N and P deficiency are gradually being revealed. Nevertheless, the adaptive responses in apple under persistent P and/or N deficient conditions have not yet been studied systematically.

Rootstock plays an important role in nutrient absorption and environmental adaptability regulation in apple (Wang et al., 2019b). Dwarfing rootstock ‘M9-T337’ delivers an early and high cumulative yield and increased fruit quality, without the need for labor-intensive inputs, and it has become a commonly used rootstock in the main apple-producing regions of China (Li et al., 2018). However, apple with ‘M9-T337’ rootstock is vulnerable to N and P nutritional deficiencies (Xie et al., 2022a; Xie et al., 2022b), because greater amounts of N are required by perennial woody plants, and the availability of inorganic P in soils is low (Carranca, 2012; Rennenberg and Herschbach, 2013). Therefore, understanding the mechanism of dwarfing rootstock ‘M9-T337’ responses to N and/or P deficiency, and thus exploring the improvement of nutrient use efficiency is of great significance for realizing sustainable development of apple in modern agriculture.

In this study, using hydroponically-grown dwarfing rootstock ‘M9-T337’ as the experimental material, we explored the variation in plant growth, root morphology, and N and P absorption under N and/or P deficiency and identified the key genes and metabolites associated with root morphological changes under different N and P supply conditions. Furthermore, we investigated the potential roles of two cell wall expansin genes, *MdEXPA4* and *MdEXLB1*, which are significantly induced by N and/or P deficiency, in root development using stable overexpression systems. Our results contribute to the understanding of root architecture regulation in apple dwarfing rootstock and provide important information about the molecular mechanisms underlying the coordinated utilization of N and P under N and P nutritional deficit environment.

## 2 Materials and methods

### 2.1 Plant material, growth conditions and treatments

Tissue-cultured ‘M9-T337’ seedlings were transplanted to soil after rooting and grown for an additional 5 weeks in a greenhouse at 25°C (day) and 20°C (night), relative humidity 65–70%, light intensity 400 μM m^−2^ s^−1^, and a photoperiod of at 16 (light):8 h (dark), using full spectrum LED lamps. During this period, plants were watered weekly with modified 1/2-strength Hoagland nutrient solution [2.5 mM (NH4)_2_Suc, 0.1 mM KH_2_PO_4_, 5.9 mM KCl, 1 mM CaCl_2_·2H_2_O, 2 mM MgSO_4_, 0.1 mM Fe-EDTANa_2_, 0.05 mM H_3_BO_3_, 0.001 mM ZnSO_4_, 0.001 mM CuSO_4_·5H_2_O, 0.012 mM MnSO_4_·H_2_O, and 0.0002 mM Na_2_M_O_O_4_·7H_2_O) to ensure normal growth. Then, 180 plants of similar size with 8–9 leaves were selected to pre-culture in deionized water for about 10 d, allowing them to adapt to the hydroponic conditions and fully consume their stored nutrients. Next, the plants were divided randomly into four groups and separately exposed to (1) N+P-sufficient (NNNP, 5 mM KNO_3_ and 1 mM KH_2_PO_4_), (2) P-deficient (NNLP, 5 mM KNO_3_ and 0.001 mM KH_2_PO_4_), (3) N-deficient (LNNP, 0.2 mM KNO_3_ and 1 mM KH_2_PO_4_), and (4) N+P-deficient (LNLP, 0.2 mM KNO_3_ and 0.001 mM KH_2_PO_4_) conditions. The four nutrient solutions each contained 1 mM CaCl_2_·2H_2_O, 2 mM MgSO_4_, 0.1 mM Fe-EDTANa_2_, 0.05 mM H_3_BO_3_, 0.001 mM ZnSO_4_, 0.001 mM CuSO_4_·5H_2_O, 0.012 mM MnSO_4_·H_2_O, and 0.0002 mM Na_2_M_O_O_4_·7H_2_O, and the pH was adjusted to 5.9. In the NNLP, LNHP, and LNLP treatments, KCl was used to replace KH_2_PO_4_ or KNO_3_ to avoid K deficiency. In this experiment, 15 hydroponic boxes (1 L volume) were set up for each treatment. Each box contained 1 L of nutrient solution and three seedlings. The nutritional treatments were continued for 8 weeks and refreshed every 7 d. A submersible pump (power: 3W) was used to supply oxygen to the nutrient solution in each hydroponic box, and 30 minutes every two hours. For ^15^N determination, nine seedlings from each treatment were selected to treat with nutrient solutions that replaced potassium nitrate with ^15^N-KNO_3_ (10.14%, Shanghai Research Institute of Chemical Industry, China).

### 2.2 Measurement of root morphology

After treatment, the roots were dispersed in water to separate them, and photographs were taken using a photo scanner (Epson, Japan). The images were analyzed using WinRHIZO Pro2009 (Regent Instruments, Canada) to determine the root parameters. Three biological replicates were assessed for each treatment.

### 2.3 Measurement of plant fresh and dry weights

The roots, stems, and leaves of three experimental replications from each treatment were harvested and the fresh weights measured. The components were then dried in an oven individually at 105°C for 30 min and then at 70°C to a constant weight. The dry weight was then measured.

### 2.4 Measurement of total N, ^15^N, and total P contents

The roots, stems, and leaves of ‘M9-T337’ seedlings labelled with ^15^N were washed three times with distilled water, fixed at 105°C for 30 min, and dried at 70°C to a constant weight before being ground into a powder. Then, 0.3 g of powdered material from each tissue type was digested using H_2_SO_4_-H_2_O_2_ and fixed in distilled water to a final volume of 50 mL. Five milliliters of the clear supernatant were removed to determine the total P content using the vanadium molybdate yellow colorimetric method (Zhang et al., 2020). The remaining 0.1 g of powdered material from each tissue type was used to determine the total N content and ^15^N abundance using a Flash 2000HT elemental analyzer coupled to a Finnigan DELTA V Advantage isotope ratio mass spectrometer (Thermo Fisher Scientific, Germany). The total N contents or ^15^N contents of the leaf, stem, and root were calculated by multiplying the N concentration or ^15^N concentration by the dry weight, respectively. ^15^N utilization efficiency (^15^NUE) was calculated as the ratio of the total ^15^N content in the seedling to the total ^15^N in the fertilizer.

### 2.5 Determination of H^+^ and NO_3_- flux

On Day 7 of the experimental period, three seedlings from each treatment were used for ion flux measurements. The net H^+^ and ${\text{NO}}_{3}^{\;-}$ flux in the rhizosphere was measured with a noninvasive micro test technique (NMT 100 Series, Younger USA LLC, USA). Ionic flux data were calculated using Mage Flux (http://xuyue.net/mageflux).

### 2.6 Metabolite profiling

On Day 30 of the experimental period, the roots from each treatment were harvested and divided into two portions, one for widely targeted metabolomics determination and the other for transcriptome sequencing. Extraction, derivatization, detection, quantification, and data analyses were carried out by Wuhan MetWare Biotechnology Co., Ltd., China. Three biological replicates were assessed for each treatment, and one biological replicate was used for every nine seedling root mixes. The metabolites were extracted with a 70% methanol solution from 0.1 g of the lyophilized powdered samples, and, subsequently, the sample extracts were analyzed using an UPLC-ESI-MS/MS system (Applied Biosystems 4500 Q TRAP, Germany; SHIMADZU Nexera X2, MS, Japan).

The metabolites in the different samples were analyzed based on the Kyoto Encyclopedia of Genes and Genomes (KEGG) compound database, MetWare database (MWDB), and multiple reaction monitoring (MRM). To study metabolite accession-specific accumulation, metabolites from 12 samples were used for hierarchical clustering analysis (HCA) and orthogonal partial least squares discriminant analysis (OPLS-DA) after being log_2_-transformed and normalized. The metabolites were selected on the basis of the combination of a statistically significant threshold of variable influence in projection (VIP) values obtained from the OPLA-DA model and *P* values from a two-tailed Student’s *t*-test on the normalized peak areas from different groups, where metabolites with VIP > 1.0 and *P<* 0.05 were considered differentially accumulated metabolites (DAMs). Venn diagrams were used to illustrate the number of DAMs among different treatments. In order to study the trend of relative metabolite content under different N and P supply conditions, the relative content of all DAMs was standardized to z-core, and then Kmeans cluster analysis was conducted based on z-core. Z-core was calculated by equation (1). x represents the quantitative value of a specific metabolite, μ represents the average quantitative value of all metabolites, and σ represents the standard deviation.


(1) $$z=\frac{\left(x-\mu \right)}{\sigma }$$

### 2.7 RNA extraction and transcriptomic sequencing

Total RNA of the samples was extracted with an RNAprep Pure Plant kit (DP441, Tiangen, China). The high-quality mRNA was randomly fragmented. First-strand cDNA was synthesized using the M-MuLV reverse transcriptase system. The RNA strand was then degraded by RNase H, and second-strand cDNA was synthesized using DNA polymerase. The double-stranded cDNAs were ligated to sequencing adapters. After amplification and purification, cDNA libraries were obtained and sequenced using a Novaseq6000 system (Illumina, USA).

Gene expression levels were determined using the RPKM (reads per kb per million reads) method. Differential expression analysis was performed using DESeq2 with a design formula [|log_2_(FoldChange)|≥ 1 and false discovery rate (FDR)< 0.05] that took into account the contrast among samples from four treatments. The genes/transcripts with an adjusted *P* value of<0.05, using multi-testing error correction (Benjamini-Hochberg test) and corresponding to a false discovery rate (FDR) of 1%, were considered to be differentially expressed genes (DEGs). All DEGs were mapped to pathway terms in the KEGG database (http://www.genome.jp/kegg/). The FPKM values after centralization and standardization were used for DEGs hierarchical clustering analysis, and the clustering heatmap was drawn.

Quantitative values of genes and metabolites in all samples were used for correlation analysis using R (corrplot, Version 0.84). Correlation results with correlation coefficient greater than 0.8 and p value less than 0.05 were selected to draw nine-quadrant heatmap and correlation heatmap.

### 2.8 Real-time RT-PCR analysis

Purified RNA was reverse transcribed to first-strand cDNA with a PrimeScript™ RT Master Mix cDNA Reverse Transcription Kit (Takara, China). qRT-PCR was conducted with a ChamQ SYBR qPCR Master Mix kit (Vazyme, China) and a C1000 Touch™ Thermal Cycler system (Bio-Rad, USA). Relative transcript levels were calculated according to the 2^−ΔΔCp^ method using *MdActin* as a reference. Three biological and technical replications were performed. The primes sequences are shown in **Table S1**.

### 2.9 Plasmid construction, genetic transformation, and functional verification

The open reading frames of *MdEXPA4* and *MdEXLB1* were respectively inserted into the pRI 101-AN vector (TaKaRa, China), and the recombinant plasmids were respectively transformed into *Agrobacterium* strain GV3101. The recombinant plasmids were transformed into Col-0 using floral-dip transformation. T0 seeds were sown on 1/2 MS medium containing Kanamycin (50 mg/L) for transgenic selection. Two homozygous T3 transgenic *A. thaliana* lines (#1 and #2) were selected for further study. The *MdEXLB1* plasmids were transfected into *Solanum lycopersicum* (cultivar Ailsa Craig) plants through leaf explant infection. Three third generation homozygous transgenic lines (#5, #8, and #11) were selected for further study.

Seeds of wild-type (WT) and *MdEXLB1-OE S. lycopersicum* were sown in MS medium. After sprouting, the seedlings were transplanted to soil and grown for an additional 2 weeks. Then, seedlings of similar size were randomly divided into four groups and separately exposed to N+P-sufficient, P-deficient, N-deficient, and N+P-deficient conditions, as in section 2.1. The nutritional treatments were continued for 8 weeks and refreshed every 7 d.

The seeds of wild-type and *MdEXPA4-OE A. thaliana* were sown in MS medium. After sprouting, the seedlings were grown on MS medium with different concentrations of ${\text{NO}}_{3}^{\;-}$ and ${\text{HPO}}_{4}^{\;2-}$ : (1) N+P-sufficient (NNNP, 60 mM ${\text{NO}}_{3}^{\;-}$ and 1.25 mM ${\text{HPO}}_{4}^{\;2-}$ ), (2) P-deficient (NNLP, 60 mM ${\text{NO}}_{3}^{\;-}$ and 0.01 mM ${\text{HPO}}_{4}^{\;2-}$ ), (3) N-deficient (LNNP, 0.2 mM ${\text{NO}}_{3}^{\;-}$ and 1.25 mM ${\text{HPO}}_{4}^{\;2-}$ ), and (4) N+P-deficient (LNLP, 0.2 mM ${\text{NO}}_{3}^{\;-}$ and 0.01 mM ${\text{HPO}}_{4}^{\;2-}$ ) conditions.

### 2.10 Statistical analysis and visualization

All physiological data were graphed using GraphPad Prism v6.01 (GraphPad Software Inc., USA). Duncan’s test (*P*< 0.05) was used to analyze the statistical significance by SAS v9.0 (SAS Institute Inc. USA).

## 3 Results

### 3.1 Comparison of growth and root morphological characteristics

**Figure 1:**
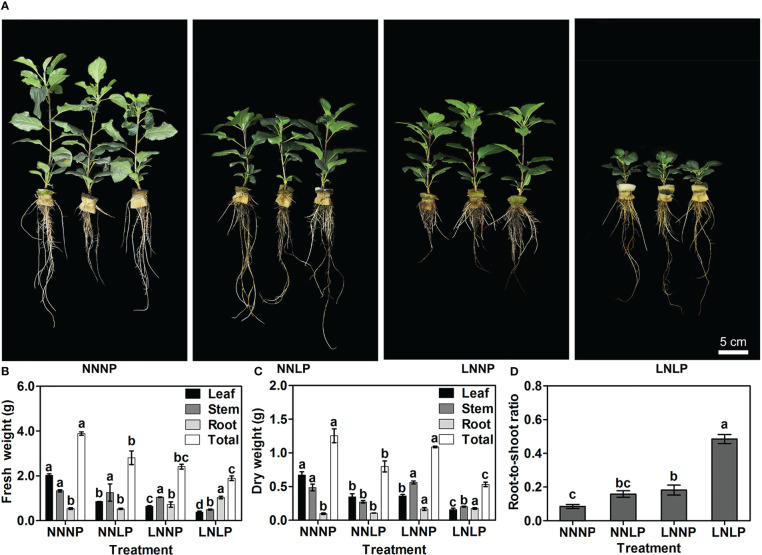
Effects of N and P supply conditions on growth of ‘M9-T337’ seedlings. **(A)** Phenotype characterization of ‘M9-T337’ seedlings after growth in N, P-sufficient (NNNP), P-deficient (NNLP), N-deficient (LNNP), and N, P-deficient (LNLP) nutrient solutions for 60 d, respectively. Scale bar = 5 cm. **(B)** Fresh weights, **(C)** dry weights and **(D)** the root-to-shoot ratio after growth in NNNP, NNLP, LNNP, and LNLP nutrient solutions for 60 d. Data were shown as means ± SE (*n* = 3), and different lowercase letters above the bars indicate significant differences among different treatments (*p*<0.05).

Compared with NNNP, the above-ground growth of ‘M9-T337’ seedlings was inhibited under NNLP, LNNP, and LNLP conditions (**Figure 1**). Relative to NNNP, the leaf fresh weight decreased by 57.64%, 68.16%, and 81.24% under NNLP, LNNP, and LNLP conditions, respectively, and the leaf dry weight decreased by 48.10%, 46.32%, and 76.49%, respectively. In addition, the stem fresh weight decreased significantly only under LNLP, whereas the stem dry weight decreased by 29.46% and 58.63% under NNLP and LNLP conditions, respectively. However, the root fresh weight increased 1.78-fold under LNLP, and the root dry weight increased 1.35- and 1.94-fold under LNNP and LNLP conditions, respectively. Overall, the total plant fresh weight decreased in the order NNNP > NNLP > LNNP > LNLP, while the total plant dry weight decreased by 36.42% and 57.55% under NNLP and LNLP, respectively (**Figures 1B, C**). Based on the dry weights of the shoot and root, 1.86-fold, 2.14-fold, and 5.71-fold increases were found in the root-to-shoot ratio under NNLP, LNLP, and LNLP conditions, respectively (**Figure 1D**).

**Figure 2:**
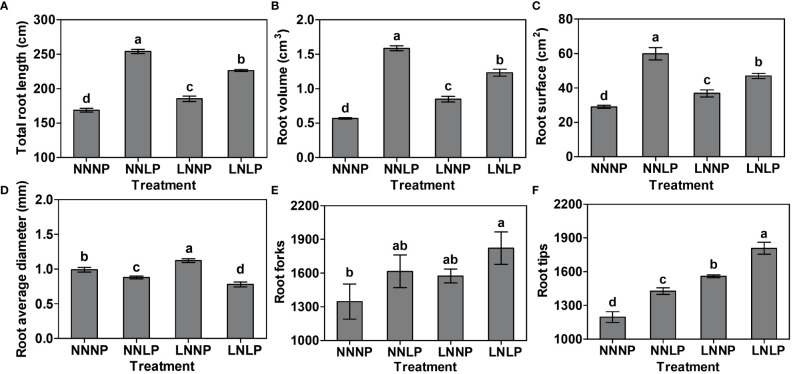
Differences in the root parameters of the "M9-T337" seedlings after growth in NNNP, NNLP, LNNP, and LNLP nutrient solutions for 60 d. **(A)** Total root length, **(B)** root volume, **(C)** root surface area, **(D)** root average diameter, **(E)** number of root forks and **(F)** number of root tips. Data are shown as means ± SE (n = 3), and different lowercase letters above the bars indicate significant differences among the treatments (p < 0.05).

Taking into account the root parameters, NNLP, LNNP, and LNLP significantly affected the total root length, volume, surface area, average diameter, and number of tips. The total values for root length, volume, and surface area decreased among treatments in the following order: NNLP > LNLP > LNNP > NNNP, and the differences were significant (**Figures 2A–C**). The root average diameter increased 1.13 times under LNNP, whereas it reduced by 11.36% and 24.40% under NNLP and LNLP, respectively (**Figure 2D**). The greatest increase in root forks was found under the LNLP condition (**Figure 2E**). Additionally, 19.20%, 25.33%, and 39.18% increases in root tips were observed in NNLP, LNNP, and LNLP, respectively (**Figure 2F**).

### 3.2 Differences in N and P absorption

**Figure 3:**
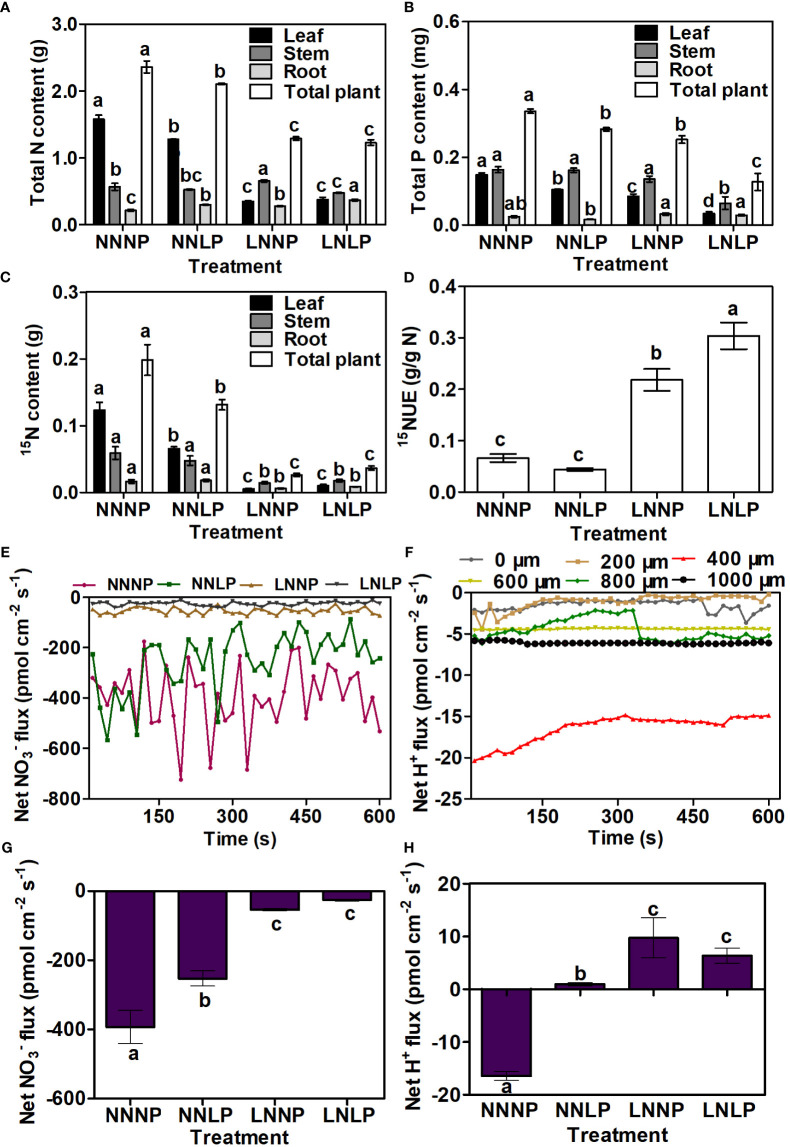
N and P absorption of ‘M9-T337’ seedlings after growth in NNNP, NNLP, LNNP, and LNLP nutrient solutions for 60 d. **(A)** Total N content and **(B)** total P in the leaf, stem, and root were measured, the content in total plant was calculated as the sum of the content in three parts. ^15^N content in the leaf, stem, root and the plant were shown as **(C)**, and ^15^NUE was calculated as the ratio of total ^15^N content in the seedling to total ^15^N in the nutrient solutions **(D)**. **(E, G)** Net fluxes of NO_3-_ after growth in NNNP, NNLP, LNNP, and LNLP nutrient solutions for 7 d. **(F)** Net fluxes of H^+^ in different regions of root surface of ‘M9-T337’ seedlings after growth on NNNP nutrient solution for 7 d. **(H)** Changes of H^+^ fluxes at 400 μm from the apical end of the root tip in roots of ‘M9-T337’ seedlings after growth on NNNP, NNLP, LNNP, and LNLP nutrient solutions for 7 d. Data in the **(A-D, G, H)** were shown as means ± SE (*n* = 3), data in the **(E-H)** positive values indicate iron influx, negative values indicate iron efflux. Duncan’s test (*p* < 0.05) was used to analyze the statistical significance, and different lowercase letters indicate significant differences among different treatments.

Compared with that in NNNP, the total leaf N contents in NNLP, LNNP, and LNLP were significantly reduced by 18.87%, 77.67%, and 75.94%, respectively; the total stem N content was increased 1.15-fold in LNNP, but reduced by 15.87% in LNLP; and the total root N contents in NNLP, LNNP, and LNLP were increased 1.39-fold, 1.30-fold, and 1.72-fold. Based the total N content in all organs, the ranking for the total N content in whole plants under different treatments was NNNP > NNLP > LNNP ≌ LNLP (**Figure 3A**). Similarly, compared to those grown under NNNP conditions, the ^15^N contents under NNLP, LNNP, and LNLP conditions were reduced by 46.87%, 95.39%, and 91.75% in leaf, respectively. In stem and root, the ^15^N contents decreased significantly under LNNP and LNLP, but the greatest decreases were found in the LNNP treatment (75.54% and 62.57%); the decreases under NNLP were not significant (**Figure 3C**). Based on the ^15^N contents of different organs, 3.30-fold and 4.60-fold increases were found in the ^15^NUE under LNNP and LNLP, but no difference was found under NNLP (**Figure 3D**). NNLP caused a reduction of ${\text{NO}}_{3}^{\;-}$ influx in comparison with NNNP, while there was much less ${\text{NO}}_{3}^{\;-}$ influx under LNNP and LNLP in comparison with NNNP and NNLP (**Figures 3E, G**).

The total leaf P content by treatment decreased in the order: NNNP > NNLP > LNNP > LNLP, and the differences were significant. The greatest decrease in the total stem P content was observed in the LNLP treatment (64.03%). Relative to NNNP, the total root P content decreased by 31.80% under NNLP but increased by 29.90% and 16.83% under LNNP and LNLP, respectively. The total P contents in whole plants under NNLP, LNNP, and LNLP decreased, with the greatest decrease found in the LNLP treatment (62.08%) (**Figure 3B**). The maximum H^+^ influx was detected at 400 μm from the apical end of the root tip in roots of ‘M9-T337’ seedlings after growth under NNNP conditions for 7 d (**Figure 3F**). However, obvious H^+^ efflux was detected in the same region of the roots of ‘M9-T337’ seedlings under NNLP, LNNP, and LNLP treatments. The maximum H^+^ efflux was detected in the roots of ‘M9-T337’ seedlings under the LNNP treatment (**Figure 3H**).

### 3.3 Assessment of metabolic changes

**Figure 4:**
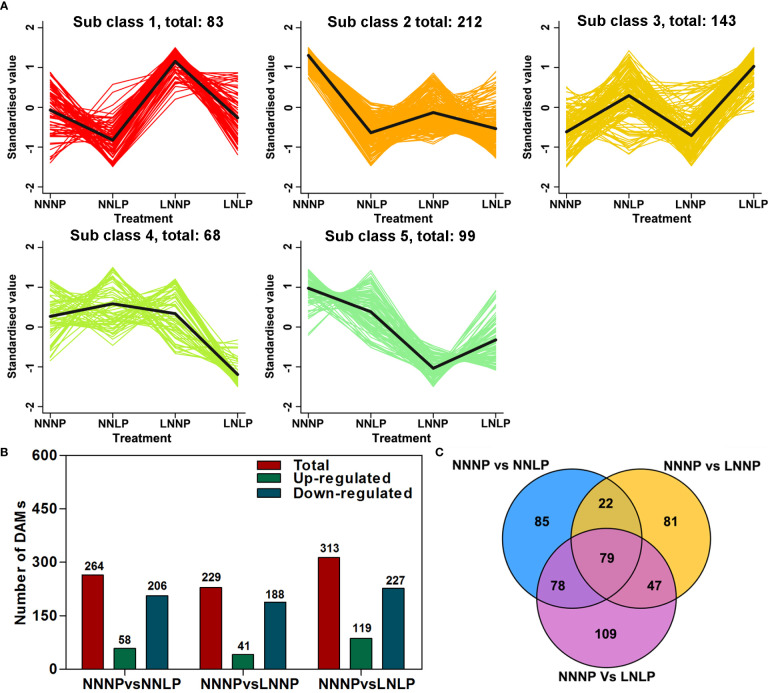
Differential metabolite analysis in the roots of ‘M9-T337’ seedlings after growth on NNNP, NNLP, LNNP, and LNLP nutrient solutions for 30 d. **(A)** K-means cluster diagram of differentially accumulated metabolites (DAMs). The X-coordinate represented the sample, the Y-coordinate represented the relative standardized metabolite content. Sub class represented the K-means clustering category number of metabolites with similar trends, and total represented the numbers of metabolites in the sub class. **(B)** Number of up-regulated, down-regulated, and total DAMs in multiple pairwise comparisons of NNNP vs. NNLP, NNNP vs. LNNP, and NNNP vs. LNLP. **(C)** Venn diagram of DAMs in multiple pairwise comparisons of NNNP vs. NNLP, NNNP vs. LNNP, and NNNP vs. LNLP.

A total of 1,069 metabolites were obtained, and these substrates were classified into 11 categories at Class 1 level; specifically: 216 flavonoids, 227 phenolic acids, 152 lipids, 96 amino acids and derivatives, 85 organic acids, 69 nucleotides and derivatives, 57 alkaloids, 41 triterpenes, 40 lignans and coumarins, 19 tannins, and 97 others (**Figure S1**). To study the metabolite accumulation patterns, a K-means cluster analysis was performed. The results showed the metabolites were divided into five subclasses (**Figure 4A**). Metabolites in subclass 1 showed an increasing trend under LNNP, and the content of metabolites in subclass 3 tended to increase under NNLP and LNLP compared with NNNP, while subclass 4 metabolites showed a decreasing trend under LNLP, and those in subclasses 2 and 5 exhibited a decreasing trend under NNLP, LNNP, and LNLP in comparison to NNNP. Compared with NNNP, 264 DAMs were observed under NNLP, of which 21.97% were significantly up-regulated and 78.03% exhibited significant down-regulation. The 313 DAMs were observed under LNLP, of which 38.02% were up-regulated and 72.52% were down-regulated. In addition, 229 DAMs were observed under LNNP, of which 17.90% were up-regulated and 82.10% were down-regulated (**Figure 4B**). Venn analysis revealed 79 common DAMs in the comparisons of NNNP vs NNLP, NNNP vs LNNP, and NNNP vs LNLP. In addition, 78 DAMs were shared in comparisons of the NNNP vs NNLP and NNNP vs LNLP treatments, 22 were shared in the comparisons of NNNP vs NNLP and NNNP vs LNNP treatments, and 47 were shared in the comparisons of NNNP vs LNNP and NNNP vs LNLP treatments (**Figure 4C**).

### 3.4 Transcriptome analysis

**Figure 5:**
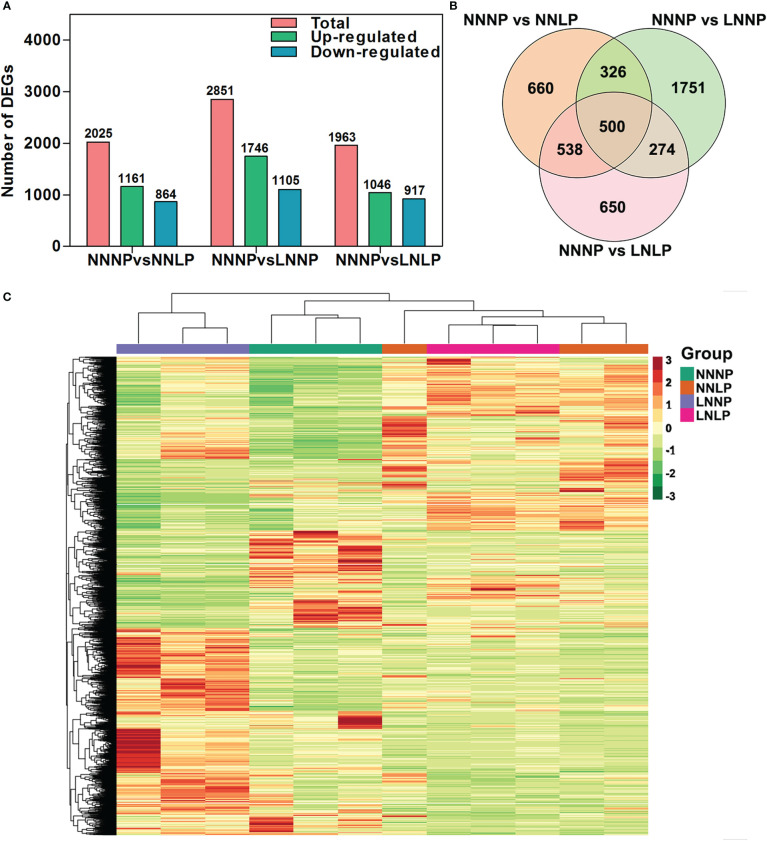
.Differential expressed gene analysis in the roots of ‘M9-T337’ seedlings after growth on NNNP, NNLP, LNNP, and LNLP nutrient solutions for 30 d. **(A)** The number of differentially expressed genes (DEGs) in the comparison of NNNP vs NNLP, NNNP vs LNNP, and NNNP vs LNLP. **(B)** Venn diagram showing the overlap among DEGs in each of the different conditions of N and P supply. **(C)** Heatmap showing the divergence of the respective transcriptome in response to N and/or P deficiency. Hierarchical clustering analysis was based on Euclidean distance among samples.

To further analyze genome-wide changes in gene expression, the 12 samples were used to construct RNA libraries and perform RNA-seq. A total of 2025 DEGs were identified in the NNNP vs NNLP comparison, of which 52.40% were up-regulated and the rest were down-regulated; a total of 2851 genes were differentially expressed in the NNNP vs LNNP comparison, of which 61.24% were up-regulated, and the rest were down-regulated; a total of 1963 genes exhibited significant differences in expression in the NNNP vs LNLP comparison, of which 52.29% were up-regulated, and the rest were down-regulated (**Figure 5A**). Five hundred DEGs were common in the comparisons of NNNP vs NNLP, NNNP vs LNNP, and NNNP vs LNLP (**Figure 5B**). Compared with NNNP, the changes in gene transcription levels under NNLP and LNLP were greater than those under LNNP (**Figure 5C**).

### 3.5 Combined transcriptome and metabolome analysis

**Figure 6:**
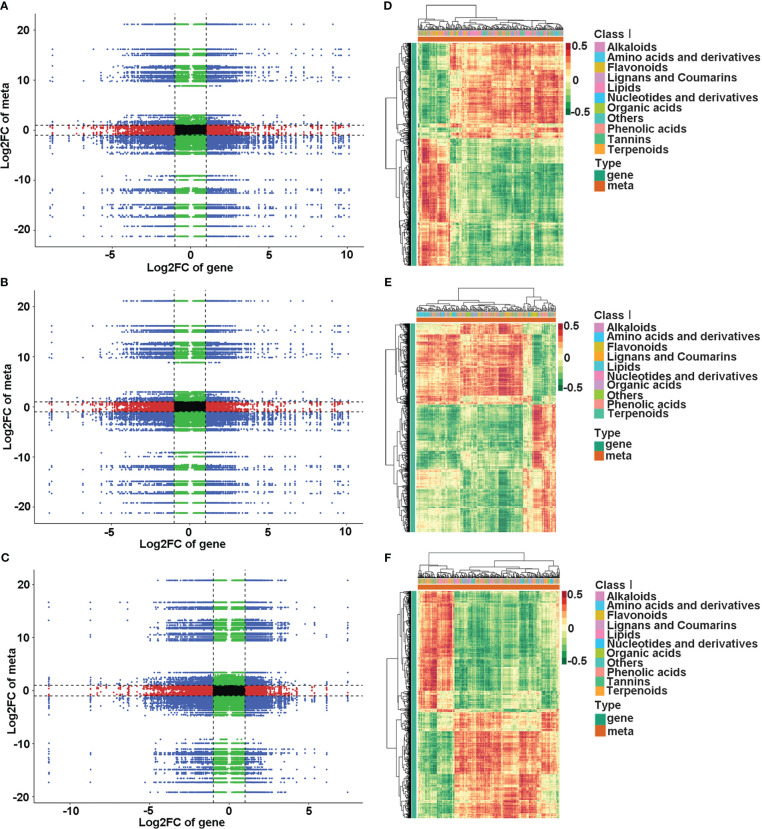
Conjoint analysis of DEGs and DAMs in the roots of the ‘M9-T337’ under P-deficiency, N-deficiency and N and P-deficiencies. Correlation analysis of transcriptomic and metabolomic data **(A–C)** and the Pearson correlation coefficient cluster heatmap of DEGs and DAMs (PCCs > 0.8 and *p*<0.05) **(D–F)** in comparison of NNNP vs NNLP **(A, D)**, NNNP vs LNNP **(B, E)** and NNNP vs LNLP **(C, F)**.

KEGG enrichment analysis showed that DEGs and DAMs in the comparison of NNNP vs NNLP were association with 62 co-mapped pathways; those in the comparison of NNNP vs LNNP were associated with 49 co-mapped pathways; and those in the comparison of NNNP vs LNLP were associated with 63 co-mapped pathways. Metabolism-related assignments such as metabolic pathways, biosynthesis of secondary metabolites, biosynthesis of cofactors, biosynthesis of amino acids, flavonoid biosynthesis, 2-oxocarboxylic acid metabolism, phenylpropanoid biosynthesis, carbon metabolism, and purine metabolism, as well as environmental information processing-related pathways, including ABC transporters, were conspicuously enriched pathways under N and/or P deficient conditions (**Figure S2**). According to the Pearson correlation coefficients (PCCs) for genes and metabolites detected in each sample, the metabolite changes may have been positively regulated by genes in the third and seventh quadrants, while the patterns of genes and metabolites from the first and ninth quadrants showed the opposite (**Figures 6A–C**). The DAMs and DEGs with PCCs greater than 0.8 are shown in **Figures 6D–F**. Respectively, 256 DAMs and 1,915 DEGs, 228 DAMs and 2,656 DEGs, and 306 DAMs and 1,893 DEGs were screened in the comparisons of NNNP vs. NNLP, NNNP vs. LNNP, and NNNP vs. LNLP. The DAMs under NNLP and LNLP conditions were distributed in the 11 groups of Class I, while the DAMs under LNNP were distributed among 10 groups. Notably, tannin substances such as procyanidin C2, strictinin, corilagin, and Sanguiin H1 were not screened under LNNP.

Association analysis based on the KEGG database showed that, in the comparison of NNNP vs. NNLP, the 68 DAMs and 413 DEGs with PCCs greater than 0.8 were mapped to 36 KEGG pathways; in the comparison of NNNP vs. LNNP, 59 DAMs and 521 DEGs with higher PCCs were mapped to 23 KEGG pathways in the comparison of NNNP vs. LNNP; and 80 DAMs and 360 DEGs with higher PCCs were mapped to 30 KEGG pathways in the comparison of NNNP vs. LNLP (**Figure S3**, **Table S2**). Among them, pentose and glucuronate interconversions, starch and sucrose metabolism, galactose metabolism, amino sugar and nucleotide sugar metabolism, and phenylpropanoid biosynthesis were conspicuously enriched pathways (**Figure S4**).

**Figure 7:**
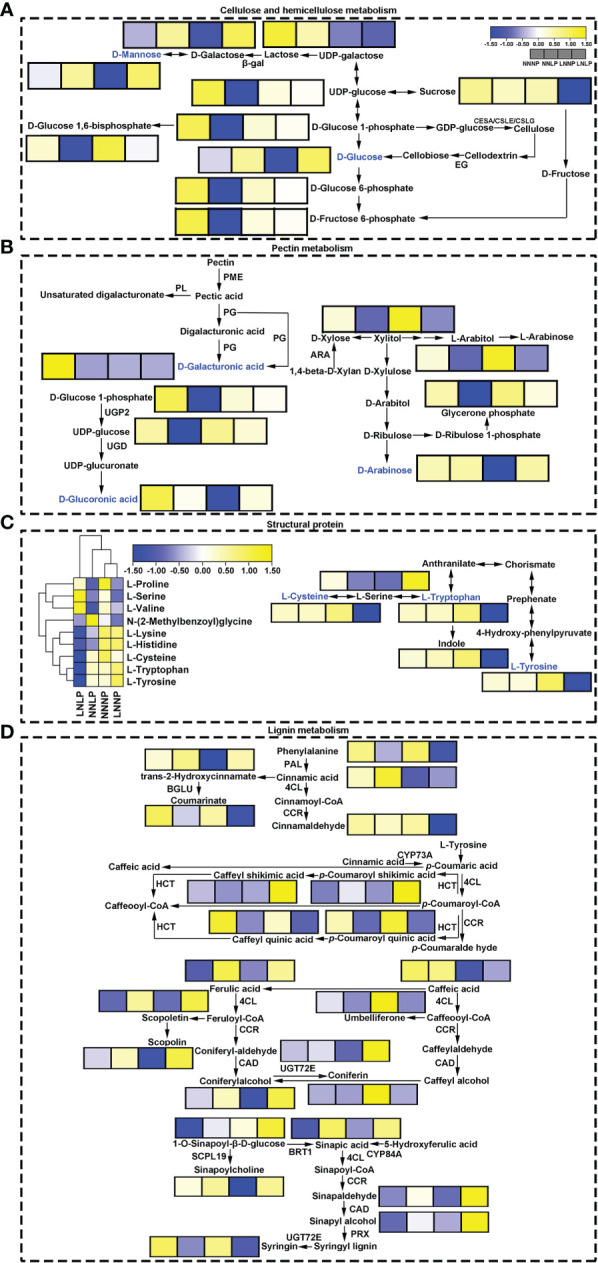
Schematic diagram showing hemicellulose metabolism **(A)**, pectin metabolism **(B)**, structural protein **(C)** and lignin biosynthesis **(D)**. The box in the pathway represents differential metabolites. Yellow and blue represent upregulated and downregulated metabolites, respectively. ARA, beta-xylosidase/alpha-L-arabinofuranosidase 1-like; β-gal, beta-galactosidase; BRT, sinapate 1-glucosyltransferase; 4CL, 4-coumarate--CoA ligase; CSLE, cellulose synthase-like E; CSLG, cellulose synthase-like G; .CAD, cinnamyl-alcohol dehydrogenase; CCR, cinnamoyl-CoA reductase; CYP73A, trans-cinnamate 4-monooxygenase; EG, endoglucanase; HCT, shikimate O-hydroxycinnamoyltransferase; PAL, phenylalanine ammonia-lyase; PG, polygalacturonase; PL, pectatelyase; PME, pectinesterase; PRX, peroxidase; SCPL, serine carboxypeptidase-like; UGD, UDP glucose 6-dehydrogenase; UGP, UTP-glucose-1-phosphate uridylyltransferase; UGT72E, coniferyl-alcohol glucosyltransferase. The same as below.

The content of D-Glucoronic acid and D-Arabinose, which involved in pectin synthesis, were reduced under LNNP, in addition, the content of Glucose-1-phosphate, Uridine 5’-diphospho-D-glucose, and D-Galacturonic acid, which related to pectin metabolism, tended to decrease under NNLP, however, the metabolites in pentose and glucuronate interconversions pathway which related to pectin metabolism did not change significantly under LNLP. The metabolites in starch and sucrose metabolism pathway and galactose metabolism pathway, and involved in hemicellulose metabolism such as D-Glucose 6-phosphate, D-Fructose 6-Phosphate, Glucose-1-phosphate, Uridine 5’-diphospho-D-glucose, D-Glucose 1,6-bisphosphate, and Dihydroxyacetone phosphate were all down-regulated under NNLP, moreover, D-Glucose 6-phosphate, D-Fructose 6-Phosphate, and Raffinose were down-regulated under LNLP, furthermore, D-Mannose and Dulcitol were down-regulated under LNNP, but D-Glucose 1,6-bisphosphate was up-regulated under LNNP. The metabolites involved in lignin metabolism pathway such as Syringin and Caffeoylquinic acid were down-regulated under NNLP and LNLP, Ferulic acid, Sinapic acid, and Sinapyl alcohol were up-regulated under NNLP and LNLP, Coumarin and Cinnamaldehyde were down-regulated under LNLP. Under LNNP, Cinnamic acid and Sinapine were down-regulated significantly. The content of amino acid that made up extension, such as L-lysine showed a decreasing trend under LNLP, and the content of amino acid that made up expansin such as L-tryptophan was decreased under LNLP. The content of above amino acids did not change significantly under NNLP and LNNP (**Figure 7**).

**Figure 8:**
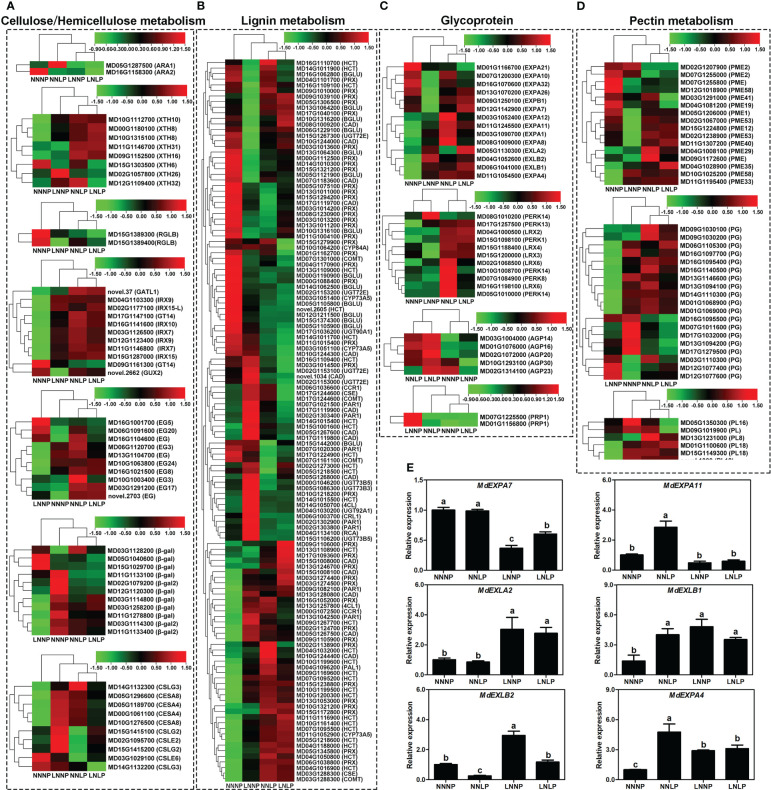
Transcript profiles of DEGs for cell wall metabolism. Heat map diagram of the log_2_ (FPKM) values for genes annotated as hemicellulose metabolism **(A)**, lignin metabolism **(B)**, glycoprotein **(C)**, pectin metabolism **(D)**, and cellulose synthase **(E)** genes. **(F)** Expression of expansin genes under different N and P supply conditions, as examined by qPCR. The transcript levels of the genes are indicated relative to the level of *MdActin* expression under NNNP (set at 1) in the same samples. Each column represents the mean ± SE of three replicates. AGP, arabinogalactan protein; BGLU, beta-glucosidase; CESA, cellulose synthase; COMT, caffeic acid 3-O-methyltransferase/acetylserotonin O-methyltransferase; CYP84A, ferulate-5-hydroxylase; EXPA, expansin A; EXPB, expansin B; EXLA, expansin-like A; EXLB, expansin-like B; GT, glycosyltransferase; GATL, galacturonosyltransferase-like; GUX, UDP-glucuronate xylan alpha-glucuronosyltransferase; HCT, shikimate O-hydroxycinnamoyltransferase; IRX, irregular xylem; LRX, leucine-rich repeat/ extension; PEX, leucine rich extensin protein; PERK, proline-rich extension-like receptor kinase; PRP, prolinerich protein; RGLB, rhamnogalacturonan endolyase; XTH, xyloglucan endotransglucosylase/hydrolase.

A total of 261 non-redundant DEGs encoding cell wall proteins or enzymes involved in cell wall metabolism were screened, of which 44 were involved in hemicellulose metabolism, 10 in cell wall synthesis, 129 in lignin metabolism, 44 in glycoprotein synthesis, and 41 in pectin metabolism (**Figure 8**).

Overall, 25 (20 up-regulated and five down-regulated), 27 (13 up-regulated and 14 down-regulated), and 18 (12 up-regulated and six down-regulated) genes involved in cellulose and hemicellulose metabolism were identified in the comparisons of NNNP vs. NNLP, NNNP vs. LNNP, and NNNP vs. LNLP, respectively. Five, two, and four genes encoding xyloglucan endotransglucosylase/hydrolase (XTH) were up-regulated in comparisons of NNNP vs NNLP, NNNP vs LNNP, and NNNP vs LNLP, respectively. The expression of genes encoding type II glycosyltransferases, such as *MD03G1126500* (*IRX7*), *MD11G1146800* (*IRX7*) *MD04G1103300* (*IRX9*), *MD15G1287000* (*IRX15*), *MD12G1123400* (*IRX9*), *MD15G1287000* (*IRX15*), and *MD02G1177100* (*IRX15-L*), showed an increasing trend in three comparisons, while *novel.2662* (*GUX2*) was down-regulated in the comparison of NNNP vs LNNP. Seven of ten DEGs encoding type III polysaccharide synthases, including *MD00G1061100* (*CESA4*), *MD05G1189700* (*CESA4*), *MD05G1296600* (*CESA8*), *MD10G1276500* (*CESA8*), *MD02G1095700* (*CSLE2*), *MD15G1415100* (*CSLG2*), and *MD15G1415200* (*CSLG2*), were up-regulated in the comparison of NNNP vs LNNP (**Figure 8A**).

A total of 58 (27 up-regulated and 31 down-regulated), 66 (29 up-regulated and 37 down-regulated), and 63 DEGs (28 up-regulated and 35 down-regulated) involved in lignin metabolism were screened in the comparisons of NNNP vs NNLP, NNNP vs LNNP, and NNNP vs LNLP, 13 DEGs, including *MD03G1051400* (*CYP73A5*), *MD17G1224900* (*HCT*), *MD12G1211500* (*BGLU*), *MD15G1374300* (*BGLU*), *MD07G1161100* (*COMT*), *MD07G1301000* (*COMT*), *MD03G1288300* (*COMT*), *MD17G1119700* (*CAD*), *MD00G1088400* (*PRX*), *MD02G1124700* (*PRX*), *MD02G1138900* (*PRX*), *MD03G1014200* (*PRX*), and *MD13G1011000* (*PRX*), were down-regulated in all three comparisons, and nine DEGs, *MD04G1016900* (*HCT*), *MD07G1095200* (*HCT*), *MD09G1169600* (*HCT*), *MD09G1267700* (*HCT*), *MD10G1161400* (*HCT*), *MD10G1199500* (*HCT*), *MD06G1038800* (*PRX*), *MD13G1053000* (*PRX*), and *MD15G1238800* (*PRX*), were up-regulated in all three comparisons (**Figure 8B**).

Six, two, and five expansin family genes were up-regulated in the comparisons of NNNP vs NNLP, NNNP vs LNNP, and NNNP vs LNLP, respectively. All DEGs in the comparison of NNNP vs NNLP were up-regulated; *MD11G1054500* (*EXPA4*) and *MD06G1041000* (*EXLB1*) were shared in three comparisons. The qRT-PCR results were generally consistent with the RNA-seq data (**Figure 8E**). A total of 18 extensin genes were differentially expressed, of which *MD17G1257500* (*PERK13*) was up-regulated in the three comparisons. Three genes encoding arabinogalactan protein, *MD03G1004000* (*AGP14*), *MD01G1076000* (*AGP16*), and *MD02G1072000* (*AGP20*), were up-regulated under NNLP and LNLP, but not differentially expressed under LNNP, while two genes, *MD10G1293100* (*AGP30*) and *MD02G1314100* (*AGP23*), were down-regulated under LNNP. Two genes encoding proline-rich proteins, *MD07G1225500* (*PRP1*) and *MD01G1156800* (*PRP1*), were down-regulated under LNNP (**Figure 8C**).

Three, seven, and three genes encoding pectinesterase (PME) were down-regulated in the comparisons of NNNP vs. NNLP, NNNP vs. LNNP, and NNNP vs. LNLP, respectively, of which *MD12G1018900* (*PME58*) was the common DEG. Three, one, and two genes were up-regulated in the comparisons of NNNP vs NNLP, NNNP vs LNNP, and NNNP vs LNLP, respectively. A total of seven (four up-regulated and three down-regulated), 15 (three up-regulated and 12 down-regulated), and six (two up-regulated and four down-regulated) encoding polygalacturonase (PG) were identified in the three comparisons NNNP vs. NNLP, NNNP vs. LNNP, and NNNP vs. LNLP, of which *MD12G1077600* (*PG*) was up-regulated and *MD17G1279500* (*PG*) was down-regulated in the three comparisons. Compared with NNNP, the expression profile of a gene encoding pectate lyase (PL), *MD13G1231000* (*PL8*), was up-regulated under NNLP, while three *PL* genes, *MD01G1100600* (*PL18*), *MD15G1149300* (*PL18*), *novel*.*1309* (*PL18*), were down-regulated under LNNP, and the remaining two genes, *MD05G1350300* (*PL16*) and *MD09G1019900* (*PL*), were down-regulated under LNLP (**Figure 8D**).

### 3.6 Functional analysis of *MdEXPA4* and *MdEXLB1*

Although *MdEXPA4* transcription was not induced under NNNP treatment (**Figure 8E**), this gene was expressed under the other three treatments. To clarify the role of *MdEXPA4* in the response to N and/or P deficiency, the phenotypes of wild-type (WT) and *MdEXPA4*-overexpressing *A. thaliana* subjected to NNNP, NNLP, LNNP, and LNLP conditions were analyzed. The results showed that *MdEXPA4*-overexpressing lines exhibited longer root length and more root forks than the WT when treated with NNNP (**Figures S8E, F**). Under NNLP conditions, the root lengths of transgenic lines #1 and #2 were 1.70-fold and 1.72-fold higher than that of the WT, respectively (**Figure S8E**). LNNP treatment led to 1.63-fold, 1.75-fold and 9.73-fold, 12.47-fold increases in the number of root forks and tips in transgenic lines #1 and #2, respectively, compared to the WT (**Figures S8F, G**). The LNLP condition caused the root lengths of transgenic lines #1 and #2 to increase by 1.34-fold and 1.54-fold compared to the WT, respectively, while the numbers of root tips also increased significantly (**Figures S8E, G**).

**Figure 9:**
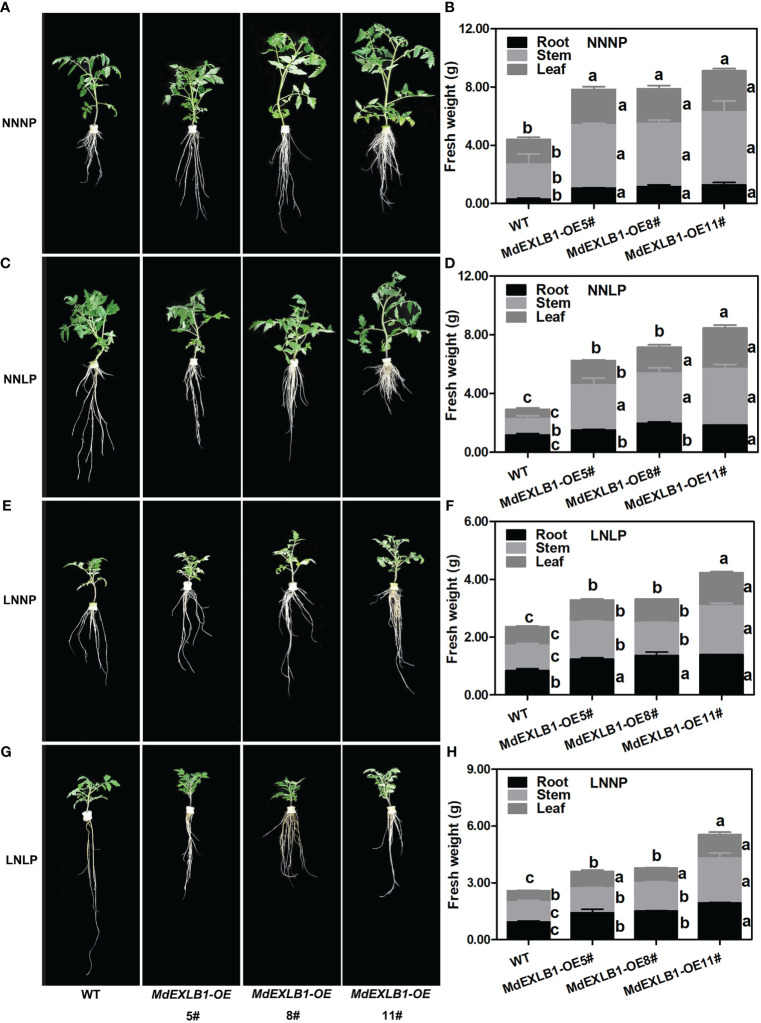
.*MdEXLB1* positively regulated N-deficiency and Pi-deficiency tolerance in tomato (*Solanum lycopersicum*). **(A, C, E, G)**, Phenotypes of *MdEXLB1* overexpressing lines subjected to NNNP, NNLP, LNNP, and LNLP conditions hydroponically for 30 days. **(B, D, F, H)**, the fresh weight of plants under different conditions. Data in **(B, D, F, H)** were shown as means ± SE (n = 3), and different lowercase letters above the bars indicate significant differences among different treatments (*p*<0.05).

Another gene, *MdEXLB1*, was hypothesized to be an important candidate for involvement in the response to N and/or P deficiency (**Figure 8E**, **Figure S10**). To evaluate the function of *MdEXLB1*, WT and *MdEXLB1*-overexpressing *S. lycopersicum* plants were cultivated hydroponically under different P and/or N supply conditions. The results showed that under the NNNP treatment, the fresh weights of transgenic lines #5, #8, and #11 were 1.78-fold, 1.79-fold, and 2.07-fold higher than that of WT, and the root fresh weights of these transgenic plants were 3.50-fold, 3.83-fold, and 4.19-fold higher than that of the WT, respectively. Under NNLP, LNNP, and LNLP conditions, the fresh weights of the transgenic plants were significantly higher than that of the WT; in particular, the roots exhibited a significant increase in fresh weight compared with those of the WT (**Figure 9**). The total length, surface area, and volume of roots in *MdEXLB1*-overexpressing lines were significantly higher than those of the WT under NNNP. Under NNLP, LNNP, and LNLP, the root surface areas of the transgenic plants were significantly higher than that of the WT (**Figure S9**). Moreover, NNNP treatment led to 1.26-fold, 1.53-fold, and 1.76-fold and 1.41-fold, 1.39-fold, and 1.28-fold increases in the total P and total N accumulation in transgenic lines #5, #8, and #11, respectively. Under the NNLP, LNNP, and LNLP treatments, the values for total P and total N accumulation were significantly higher than those in the WT (**Figure S9**).

## 4 Discussion

### 4.1 Differences in growth, root morphology and nutrient absorption of apple dwarfing rootstock in response to P and/or N deficiency

In field conditions, the availability of Pi and ${\text{NO}}_{3}^{\;-}$ is one of the most important limiting factors for apple development (Sun et al., 2021a; Sun et al., 2021b; Chai et al., 2022). The availability of these nutrients impacts the phenome, ionome, transcriptome, and metabolome composition of the plants (Gan et al., 2016; Sun et al., 2020b; Lv et al., 2021; Mu and Chen, 2021; Nezamivand-Chegini et al., 2022; Wen et al., 2022). The paramount relevance of the rootstock is its key role at the interface between the apple plants and soil for nutrient mining (Chauhan et al., 2020). In the present study, we observed that the aboveground growth and development of apple was inhibited by N and/or P deficiency (**Figure 1**), and the partitioning of carbohydrate and mineral nutrient resources to root biomass tended to increase (**Figure 3A**), thus leading to an increase in the root-to-shoot ratio of the hydroponically-grown dwarfing rootstock ‘M9-T337’ (**Figure 1D**), which was consistent with previous results (Sun et al., 2021a; Sun et al., 2021b). Generally, root development was enhanced by N and/or P deficiency, although P deficiency promoted root elongation, while N deficiency did not, and similarities existed between the root morphology under N+P deficit condition and that under P deficit and N deficit conditions, respectively, but the effect of N+P deficiency on root morphogenesis was not the accumulation of effects of P deficiency and N deficiency, and compared with P deficiency, root development was weakened under N+P deficiency (**Figures 1**, **2**). These results suggested that the ‘M9-T337’ rootstock is highly plastic and responsive to the changing soil environment (Ma et al., 2020; Tahir et al., 2021a; Tahir et al., 2021b; Zhang et al., 2021b).

Previous studies have shown that N deficiency increased H^+^-ATPase activity and H^+^ efflux, therefore affecting lateral root (LR) development and ${\text{NO}}_{3}^{\;-}$ uptake (Mlodzinska et al., 2015; Jiang et al., 2017; Lv et al., 2021). In a study of *O. sativa*, Wang et al. (2021b) found that H^+^ efflux was important for root elongation and P uptake under P deficient conditions. Moreover, Xu et al. (2020) confirmed the important role of the H^+^ pump in white lupin adaptation to P deficiency. To measure the concentration gradient of ${\text{NO}}_{3}^{\;-}$ and H^+^, while preserving the integrity of the sample, NMT was used in this study (Huang et al., 2017; Lv et al., 2021). The ionome data indicated that, although the reduced net ${\text{NO}}_{3}^{\;-}$ influx rate resulted in decreased total ^15^N accumulation in plants, N deficiency and N+P deficiency improved the ^15^NUE compared to a sufficient N+P supply (**Figure 3**). We monitored obvious H^+^ efflux in the roots under N-deficient and N+P-deficient conditions (**Figure 3H**), which resulted in a decrease in the pH of the nutrient solution (**Figure S7**). These results confirmed that H^+^ pumps stimulated by N deficiency play a crucial role in ${\text{NO}}_{3}^{\;-}$ absorption of apple rootstock. The H^+^ efflux monitored under P deficiency implied that the H^+^ pumps were key players in the response to P deficiency.

N transporters (NRT) and P transporters (PHT) play important roles in plant uptake and transport of N and P, respectively (Zhang et al., 2021c; Chai et al., 2022). NRTs in higher plants include low affinity transporters encoded by some members of the *NRT1/PTR* (also named as *NPF*) family and high affinity transporters encoded by the *NRT2* gene family (Tahir et al., 2021c; Misawa et al., 2022), as well as NRT1.1, a dual-affinity ${\text{NO}}_{3}^{\;-}$ transporter (Ye et al., 2019). Four NTR2 transporters (NRT2.1, NRT2.2, NRT2.4, and NRT2.5) and two NRT1 transporters (NPF6.3 and NPF4.6) have root ${\text{NO}}_{3}^{\;-}$ uptake activities (Lezhneva et al., 2014). In this study, 20 *NRT*s (*NRT2.5*, *NRT2*.7, and 18 *NRT1*s) were up-regulated under N deficiency, and 10 of them were also induced by N+P deficiency (**Figure S5**), and therefore may be high affinity ${\text{NO}}_{3}^{\;-}$ transporters. Lezhneva et al. (2014) reported that *NRT2.5* plays an essential role in response to severe N deficiency. *NRT2*.7 is a seed-specific high-affinity nitrate transporter controlling ${\text{NO}}_{3}^{\;-}$ content in *A. thaliana* (David et al., 2014; Zheng et al., 2016). In this study, it was found that *NRT2.5* and *NRT2*.7 may be involved in ${\text{NO}}_{3}^{\;-}$ uptake of ‘M9-T337’ root under N deficiency and P deficiency, respectively (**Figure S5**). *AtNRT1.5* drives root-to-shoot transport of ${\text{NO}}_{3}^{\;-}$ and is also an indole-3-butyric acid transporter, involved in LR development (Watanabe et al., 2020; Chen et al., 2021). Gho and Jung (2019) found that *AtNRT1.5* was induced by Pi deficiency in *A. thaliana*; they demonstrated that *atnrt1.5* mutants displayed conspicuously longer PRs along with significantly reduced LR density under P deficient conditions. Therefore, *NRT1.5* may also be involved in apple root architecture modification under N and/or P deficient conditions. Chai et al. (2022) found overexpression of *MdNRT2.4* enhanced net H^+^ extrusion from the root surface and ${\text{NO}}_{3}^{\;-}$ absorption. In this study, three *MdNRT2.4* genes were only significantly upregulated under N+P deficient condition; thus, they may have important roles in N absorption under combined nutritional deficiencies. Additionally, 19 *NRT1*s were down-regulated in the root of ‘M9-T337’ under N deficiency, and 11 of them were also down-regulated under N+P-deficient conditions (**Figure S5**), implying they may be low affinity transporters, which do not act at low ${\text{NO}}_{3}^{\;-}$ concentrations (Sinha et al., 2020). Similarly, Hu et al. (2011) found that plants repress N assimilation and save more energy for Pi acquisition under P deficiency (**Figure S5**), which is in agreement with our results. The low affinity ${\text{NO}}_{3}^{\;-}$ transporters were down-regulated in response to P deficiency (Nezamivand-Chegini et al., 2022).

Seven *PHT1s* were upregulated in response to P deficiency and N+P deficiency (**Figure S5**). The PHT1 subfamily contains high affinity Pi transporters, which function under low P concentrations in soil (Gho and Jung, 2019). Moreover, *SPX1*, *SPX3*, and *SPX5* were up-regulated under P deficiency and N+P deficiency (**Figure S5**). Studies have shown that most SPX subfamily members are induced by Pi deficiency in plants (Stefanovic et al., 2007; Zhang et al., 2016; Du et al., 2017; Wang et al., 2021a), and their major role is to interact with PHR1, thereby regulating the transcription of downstream PSIs (Lv et al., 2014; Dong et al., 2019). Furthermore, *PHO1* was down-regulated under three treatments. According to previous studies, its homologs AtPHO1, AtPHO1;H1, and OsPHO1;2 are involved in regulating internal Pi homeostasis by transporting Pi from the root xylem to shoots (Secco and Poirier, 2010; Stefanovic et al., 2011), implying the translocation of Pi from the roots to shoot is inhibited by N and/or P deficiency. The *PHT*s exhibited a lower transcript level under N+P deficiency, and five *PHT*s (two *PHT1* and three *PHT4* genes) were down-regulated under N deficiency. The expression of SPXs was affected by N availability, it was speculated that ${\text{NO}}_{3}^{\;-}$ deficiency may posted a negative effect on P absorption in apple dwarfing rootstock (Hu et al., 2019; Medici et al., 2019; Zhang et al., 2021e).

### 4.2 Genes and metabolites associated with cell wall reprogramming were activated under N and/or P deficiency

The number of DAMs specifically responding to N deficiency and P deficiency was fewer than the number responding to N+P deficiency (**Figure 4**). The number of DEGs responding specifically to P deficiency and N+P deficiency was almost same, but fewer than the number that responded to N deficiency (**Figure 5**). Thus, greater disturbance to metabolites occurred in response to combined nutritional deficiencies than a single nutrient deficiency, and the molecular mechanisms in response to combined nutritional deficiencies were rather complex. Several pathways involved in cell wall reprogramming were conspicuously enriched. However, ‘M9-T337’ adapts it root development differently under N and/or P deficiency, due to the variation of metabolites related to cell wall synthesis was different (**Figure S2**). The plant cell wall is a dynamic network composed of cellulose, hemicellulose, pectin, lignin, and multiple types of structural proteins (Lampugnani et al., 2018). Under N deficiency, the plants altered their cell wall organization and adapted their root architecture by elongating the LRs to maximize N acquisition (Sun et al., 2017a). Kumar et al. (2021) revealed that cell wall reorganization, and associated activity-related gene up-regulation, is a strategy for tolerating P deficiency in *O. sativa*. Cell wall synthesis, composition, and reprogramming contribute to the meticulous modulation of root architecture and plant adaptations to biotic and abiotic stress (Ogden et al., 2018).

Cellulose is a load-bearing polymer present in the plant cell wall (Zhang et al., 2021a). Cellulose synthase complexes (CESAs) are involved in the formation of both primary and secondary cell walls (Endler and Persson, 2011). The roles of different subunits of CESAs vary, with CESA4, CESA7, and CESA8 involved in secondary cell wall formation in *A. thaliana* (Endler and Persson, 2011). In this study, *CESA4* and *CESA8*, which are highly homologous to *AtCESA4* and *AtCESA8*, were highly induced by N deficiency (**Figure 8**). Therefore, N deficiency could increase cellulose formation in the root of ‘M9-T337’. A similar result was also reported by Nezamivand-Chegini et al. (2022). However, under P deficiency and N+P deficiency, no distinct cellulose synthesis was discerned. Hemicellulosic polysaccharides, including xylans, xyloglucans, mannans, glucomannans, and β-(1,3;1,4)-glucan, all harbor β-(1,4)-glycosyl-linked backbones with similar equatorial configurations (Zhang et al., 2021a). Xylans are the most abundant hemicellulosic polymers in vascular plants (Smith et al., 2017). IRX10 and its homolog IRX10L (members of the GT47 family), as well as IRX9, IRX9L, IRX14, and IRX14L (members of the GT43 family), are key components required for xylan backbone synthesis (Wu et al., 2010; Chen et al., 2013). IRX9, IRX10, and IRX14 are mainly involved in the extension of the backbone, while GUX1 and GUX2 are able to synthesize almost all side chains (Zhang et al., 2021a). In addition, IRX7 (a member of the GT47 family), IRX15, and its homolog IRX15L (members of the GT8 family), are also involved in xylan synthesis (Lee et al., 2009; Brown et al., 2011; Jensen et al., 2011). The transcriptome data showed that under all N deficiency, P deficiency, and N+P deficiency treatments, *IRX7*, *IRX9*, *IRX10*, *IRX15*, and its homolog *IRX15L*, were up-regulated, while the expression of *GUX2* showed a decreasing trend under LNNP. These results suggested that xylan backbone synthesis in root of ‘M9-T337’ was enhanced by N and/or P deficiency, but side chain synthesis was inhibited by N deficiency. Xyloglucan is the most abundant primary-wall hemicellulose in all spermatophytes except grasses (Zhang et al., 2021a). XTHs are involved in the rearrangement of xyloglucans by cutting and rejoining them, causing reversible or irreversible loosening of cell walls to permit cell expansion (Nezamivand-Chegini et al., 2022). Zhang et al. (2022) reported that *GmXTH38* is induced by P deficiency in both roots and leaves of soybean (*Glycine max*), and overexpression of *GmXTH38* increases the sensitivity of LR formation under P deficiency. In this study, the transcription levels of *XTHs* in roots of ‘M9-T337’ were not only induced by P deficiency and N+P deficiency, but also by N deficiency (**Figures 7A**, **8A**). These results showed that N and/or P deficiency had a role in the formation of xyloglucans in primary-wall hemicellulose of root cells, thereby mediating LR formation (Bourquin et al., 2002).

Pectins are a group of cell wall polysaccharides that possess α‐(1,4)‐linked galacturonic acids in their backbone. They play important roles in cell wall remodeling. Pectin deesterification stiffens the cell wall and prevents cell wall loosening (Hocq et al., 2017). Previous studies have shown that the enzymes involved in pectin synthesis are co-regulated together with ${\text{NO}}_{3}^{\;-}$ carriers (Landi and Esposito, 2017). In addition, pectin has been demonstrated to contribute greatly to P remobilization from the cell wall. Elevated PME activity can facilitate the remobilization of P deposited in the cell wall (Zhu et al., 2016). Wu et al. (2022) found that *OsPME14* overexpression increased PME activity, released more P from the root cell wall, and improved plant resistance to P deficiency. Zhu et al. (2018) reported that in *O. sativa*, ${\text{NO}}_{3}^{\;-}$ deficiency caused higher levels of nitric oxide to accumulate in the roots and increased the activity of nitrate reductase. The nitric oxide stimulated cell wall pectin synthesis and demethylation of pectin, therefore increasing cell wall P release by increasing PME activity under P deficiency. Unlike *O. sativa*, N and/or P deficiency were unfavourable to the biosynthesis of cell wall pectin in the root of ‘M9-T337’ (**Figures 7A**, **8A**), the effect of N deficiency was more significant since more *PME*s, *PG*s, and *PL*s were significantly down-regulated under N deficiency (**Figure 8A**), suggesting that the response mechanism is unique among different species. Nevertheless, the expression of several *PME* genes showed an increasing trend under N and/or P deficiency (**Figure 7A**), thus the release of cell wall P may be increased.

Lignin is an unordered polymer composed of phenylalanine-derived aromatic monomer substances (Vanholme et al., 2019), and it is the primary structural component of thickened secondary cell walls in vascular plants (Bonawitz and Chapple, 2010). Lignin is composed of *p*-hydroxyphenyl units without methoxyl group (synthesized by the polymerization of *p*-coumaryl), guaiacyl units with one methoxyl group (synthesized by the polymerization of coniferyl), and syringyl units with two methoxyl groups (synthesized by sinapyl alcohol) (Xie et al., 2018; Gou et al., 2019). Qin et al. (2019) reported that N deficiency reduces the activity of most peroxidases, inhibits lignin biosynthesis, and lowers root solidity. Similar results were found in present study. Most *PRX* genes which encoded peroxidase and were involved in syringyl lignin biosynthesis were down-regulated under N deficiency and the content of coniferaldehyde and coniferyl alcohol tended to decrease (**Figures 8B**, **7D**), therefore, N deficiency inhibited synthesis of guaiacyl units and syringyl units in the roots of apple dwarfing rootstock. However, P deficiency and N+P deficiency had no obvious inhibitory effect on guaiacyl synthesis. Additionally, in contrast to N deficiency, P deficiency and N+P deficiency posted a positive effect on the formation of syringyl units during lignin synthesis. Study has shown that overexpression of *Gh4CL7* promoted lignin biosynthesis in *A. thaliana*, increased lignin content in plants, and promoted root elongation, thus improved drought tolerance of plants (Sun et al., 2020a). Therefore, the up-regulated *4CL* genes and up-regulated syringyl units may be one of the important reasons that P deficiency and N+P deficiency promoted root elongation of ‘M9-T337’.

In summary, N and/or P deficiency enhanced the formation of hemicellulose xyloglucans, the extension of xylan backbone, and the loosening of cell wall. N deficiency increased cellulose formation, but inhibited the biosynthesis of hemicellulose xylan side chains, pectin and lignin. P deficiency and N+P deficiency posted a positive effect on the formation of syringyl units during lignin synthesis. These changes lead to different root morphogenesis under N and/or P deficient conditions.

### 4.3 Expansin involved in apple dwarfing rootstock morphogenesis under N and/or P deficiency conditions

Expansins are often regulated by nutrient stress to subsequently affect plant growth and nutrient uptake (Lee and Kim, 2013; Marowa et al., 2016; Kong et al., 2019). Liu et al. (2019) found that *HvEXPA1* participates in root cell elongation and influences Al content by regulating root cell wall loosening when exposed to Al stress. Wu et al. (2022) screened 40 differentially expressed expansin genes in roots of oilseed rape (*Brassica napus*) in response to boron deficiency. Similar results were also reported by Nezamivand-Chegini et al. (2022). Through analyzing the similarities in the transcriptional responses in the roots under N and/or P deficit conditions, we observed up-regulation of six, two, and five expansin genes under N deficiency, P deficiency, and N+P deficiency, respectively. The content of L-tryptophan showed an increasing trend under N deficiency (**Figure 7C**). This amino acid is not only a precursor to auxin synthesis, but is also involved in expansin protein synthesis (Dai et al., 2013; Marowa et al., 2016). Lee and Kim (2013) reported that overexpression of *EXPA17* in *A. thaliana* increased the density LRs treated with auxin. Under N deficiency, the recruitment of Thr101 phosphorylated NRT1.1 into the plasma membrane facilitates auxin transport, therefore root abundance increases under the action of expansins (**Figures 1**, **2**) (Bouguyon et al., 2015; Zhang et al., 2019). Ding et al. (2021) found that P deficiency induced the expression of multiple expansin genes in roots, related to increased IAA content caused by P deficiency, thereby promoting root lengthening, which agrees with our results. In soybean, the protein product of *GmPTF1*, the expression of which is induced by Pi deficiency, directly binds to the E-box motif in the promoter region of the cell wall relaxation gene *GmEXPB2*, influencing LR formation and elongation, thereby improving P uptake efficiency (Yang et al., 2021).

**Figure 10:**
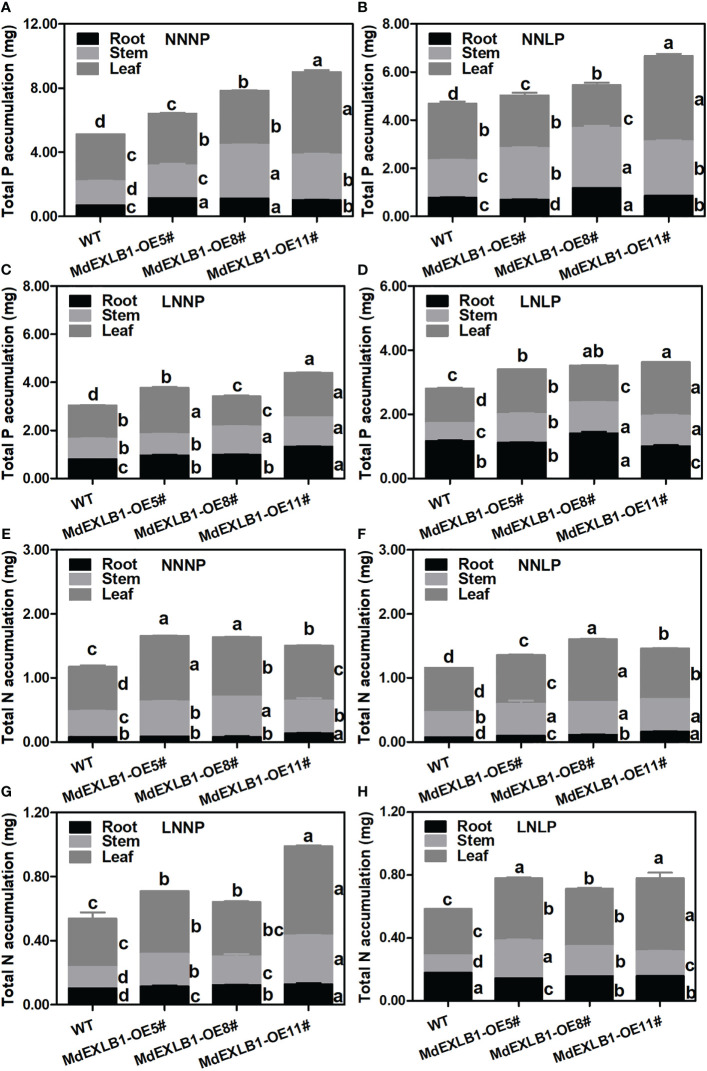
Total N **(A–D)** and total P **(E–H)** accumulation of WT and MdEXLB1 overexpressing lines under different N and P supply conditions. Data were shown as means ± SE (n = 3), and different lowercase letters above the bars indicate significant differences among different treatments (p < 0.05).

Expansins break the noncovalent bonds between cellulose microfibrils and associated matrix polysaccharides and induce extension of the cell wall (Lee and Kim, 2013; Marowa et al., 2016). A drop in pH may be one of the reasons for increasing wall extension and the activity of the wall-modifying agent expansin (**Figure S7**) (Arsuffi and Braybrook, 2018). Phylogenetic analysis showed that different expansins from various species falling within the same clade have similar effects on plant growth and development (Marowa et al., 2016). It has been reported that overexpressing *RhEXPA4* increased the abundance of LRs in transgenic *A. thaliana* plants (Lü et al., 2013). In this study, we observed similar phenotypes; overexpression of *MdEXPA4*, which is highly homologous to *RhEXPA4*, promoted *A. thaliana* LR formation and development under N+P sufficiency (**Figure S5A**). Moreover, compared to the WT, the tolerance to P deficiency and N+P deficiency was enhanced in transgenic *A. thaliana* plants. Furthermore, under N deficiency, the enhanced root system resulted in a clear benefit to the aboveground growth of the transgenic plants. Boron et al. (2015) found that overexpression of *AtEXLA* promoted root elongation. Moreover, a recent study showed that *GmEXLB1* expression was induced in soybean under P deficiency, and overexpression of this gene improved P acquisition by regulating root elongation and architecture in *A. thaliana* (Kong et al., 2019), which provided a possible direction for research on the function of this gene in apple. In this study, we confirmed that overexpression of *MdEXLB1*, which is highly homologous to *GmEXLB1* and *AtEXLA2*, altered the root architecture of transgenic *S. lycopersicum* by increasing the number and length of LRs, thereby enhancing P acquisition. Additionally, we found that *MdEXLB1* was induced by N deficiency. Overexpression of *MdEXLB1* also enhanced N acquisition and biomass accumulation, and thus promoted the tolerance of transgenic *S. lycopersicum* to N deficiency (**Figures 9**, **10**). Further understanding of the molecular mechanisms of expansin-based responses to N and/or P deficiency may lead to insights for improving nutrient utilization efficiency in apple rootstock.

## 5 Conclusion

Our research provided detailed information on the morphological, ionic, transcriptional, and metabolic responses of apple dwarfing rootstock to different N and P supply conditions. Strategies for adaptation to N and/or P deficiency in ‘M9-T337’ included inhibiting aboveground growth, enhancing root development, improving the root-to-shoot ratio, and inducing the expression of high affinity NO_3_- transporter and Pi transporter genes. Further analysis indicated that alterations to root architecture under N and/or P deficiency were associated with several factors, including: (a) increased partitioning of total N and total P in root; (b) increased H^+^ efflux rate and (c) altered root cell wall components and structrual proteins. Expansin genes *MdEXPA4* and *MdEXLB1* acted as important structural genes involved in root morphogenesis of apple dwarfing rootstock. These findings may help researchers to improve root architecture and nutrient use efficiency in apple rootstock under N and/or P deficiency conditions.

## Data availability statement

The original contributions presented in the study are publicly available. This data can be found here: NCBI BioProject PRJNA936594, accession numbers SRR23527441, SRR23527440, SRR23527439, SRR23527438, SRR23527437, SRR23527436, SRR23527447, SRR23527446, SRR23527445, SRR23527444, SRR23527443, SRR23527442.

## Author contributions

BX: Conceptualization, methodology, validation, writing—original draft preparation, data curation. YC: Conceptualization, methodology, data curation, resources, writing—review and editing, funding acquisition. YZ, AY and XL: Formal analysis. XA and CC: Supervision, project administration, funding acquisition. GK and JZ: Methodology, writing—review and editing. All authors contributed to the article and approved the submitted version.

## Supplementary material

Supplementary Figure 1 Heatmap of metabolites content in the roots of ‘M9-T337’ seedlings after growth on NNNP, NNLP, LNNP, and LNLP nutrient solutions for 30 d. Group represented samples, class represented Class 1 substances classification, the different colors were the values obtained after normalisation of the relative content (red for high content, green for low content).

Supplementary Figure 2 Histogram of KEGG classification of differential metabolites. The vertical coordinate was the name of the KEGG metabolic pathway and the horizontal coordinate was the number of metabolites annotated to that pathway and their number as a proportion of the total number of metabolites annotated on the corresponding KEGG pathway.

Supplementary Figure 3 Venn diagrams showing the overlap among DAMs and DEGs in each of the different conditions of N and P supply screened based on conjoint analysis.

Supplementary Figure 4 Bubble diagram showing the divergence of the KEGG pathways contained DAMs and DEGs in response to N and/or P deficiency screened based on conjoint analysis. The horizontal coordinate represents the enrichment factor (Diff/Background) of the pathway in different histologies, and the vertical coordinate represents the name of the KEGG pathway; the red-yellow-green gradient represents the change in the significance of the enrichment from high to medium to low, and is indicated by -log10(P value); and the size of the bubble represents the number of DAMs or DEGs, the larger the number, the larger the point.

Supplementary Figure 5 Heatmap of DEGs involved in uptake and transport of N **(A)** and P **(B)** under different N and P supply conditions.

Supplementary Figure 6 Phylogenetic relationship of expansin genes involved in root development and growth from *Arabidopsis thaliana, Oryza sativa, Hordeum vulgare, Glycine max, Rosa rugosa, Triticum aestivum, Gossypium arboretum*, and *Malus hupehensis*.

Supplementary Figure 7 Differences in pH of nutrient solution with different N and P concentrations on Day 60.

Supplementary Figure 8 Phenotypes of *MdEXPA4* overexpressing lines subjected to NNNP, NNLP, LNNP, and LNLP conditions based on MS culture medium for two weeks were showed in **A–D**, respectively. Bar=1 cm. **E–H**, the root parameters of *A. thaliana* under different N and P supply conditions.

Supplementary Figure 9 Differences in the growth and root parameters of wild-type and *MdEXLB1* overexpressing lines under different N and P supply conditions.

Supplementary Figure 10 Correlation between RNA-Seq and qRT-PCR data. Each RNA-Seq expression data was plotted against qRT-PCR data and fitted into a linear regression.

Supplementaty Table 1 Primer names and sequences.

Supplementaty Table 2 KEGG pathways mapped by differential genes and differential metabolites with higher PCCs.
