# Supplementary figures and images for: Comparative physiological, metabolomic, and transcriptomic analyses reveal mechanisms of apple dwarfing rootstock root morphogenesis under nitrogen and/or phosphorus deficient conditions
Source: Front Plant Sci. 2023 Jun 19;14:1120777. doi: 10.3389/fpls.2023.1120777 (PMC10315683; doi:10.3389/fpls.2023.1120777)

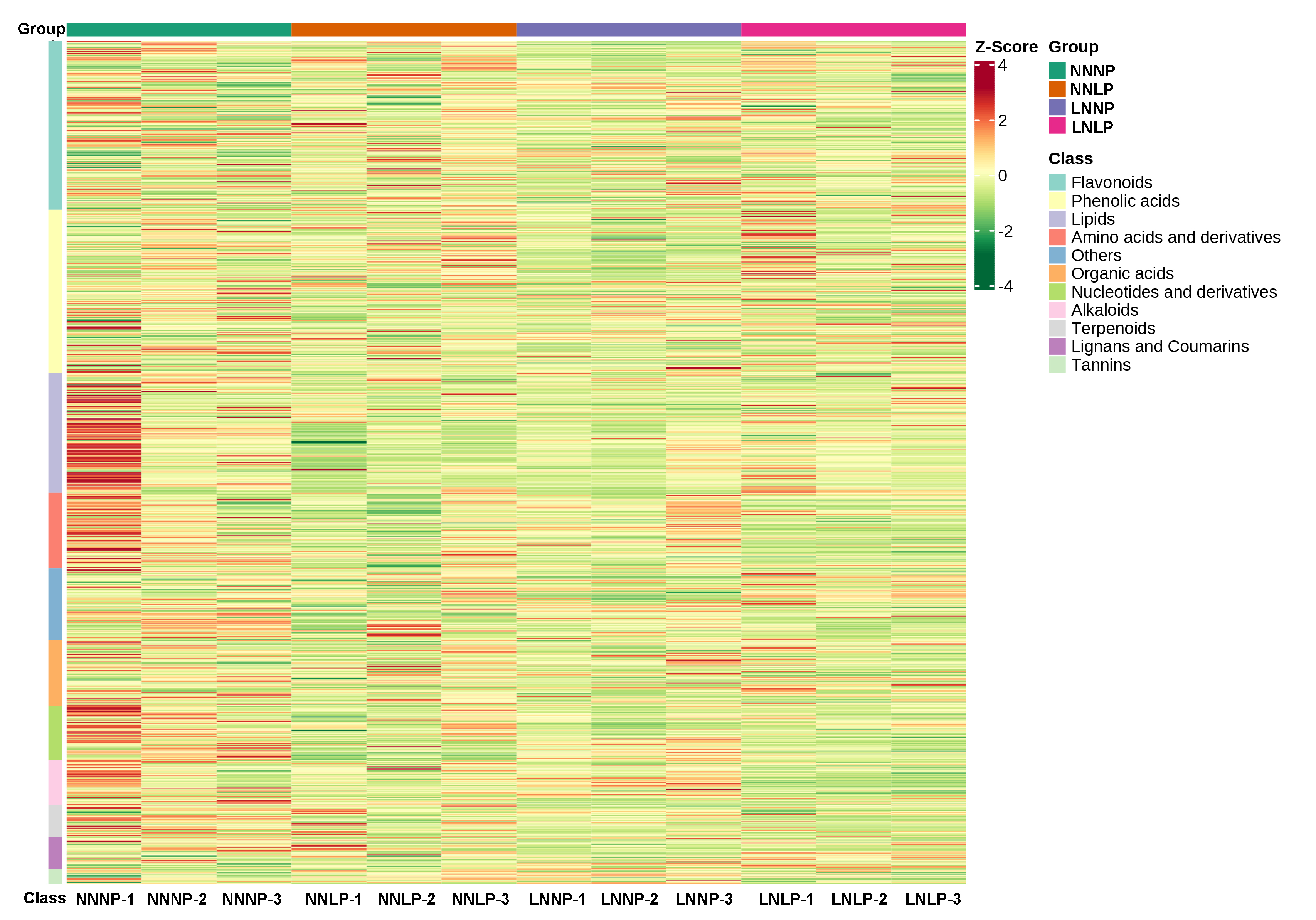

Supplement: Supplementary Figure 1 — Heatmap of metabolites content in the roots of ‘M9-T337’ seedlings after growth on NNNP, NNLP, LNNP, and LNLP nutrient solutions for 30 d. Group represented samples, class represented Class 1 substances classification, the different colors were the values obtained after normalisation of the relative content (red for high content, green for low content). [file Image_1.jpeg]

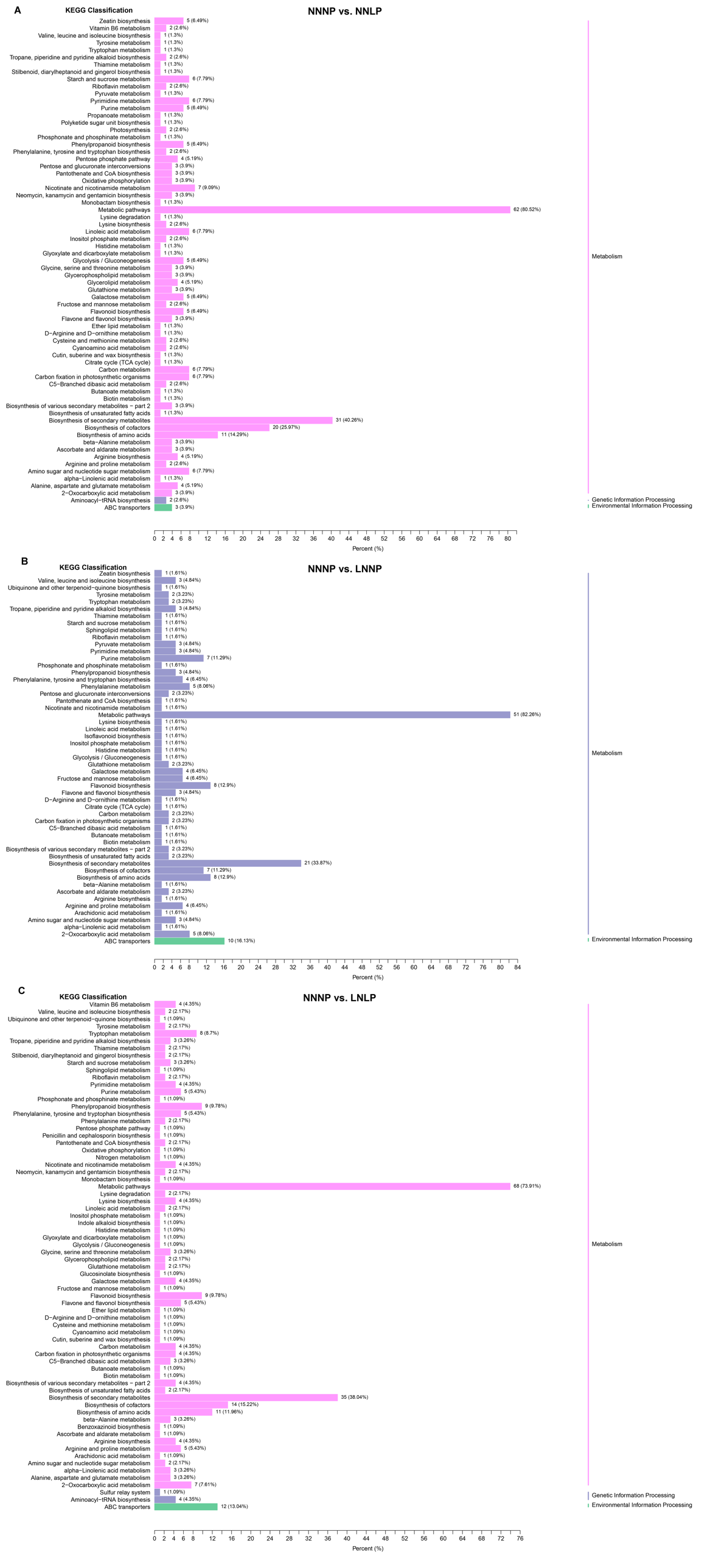

Supplement: Supplementary Figure 2 — Histogram of KEGG classification of differential metabolites. The vertical coordinate was the name of the KEGG metabolic pathway and the horizontal coordinate was the number of metabolites annotated to that pathway and their number as a proportion of the total number of metabolites annotated on the corresponding KEGG pathway. [file Image_2.jpeg]

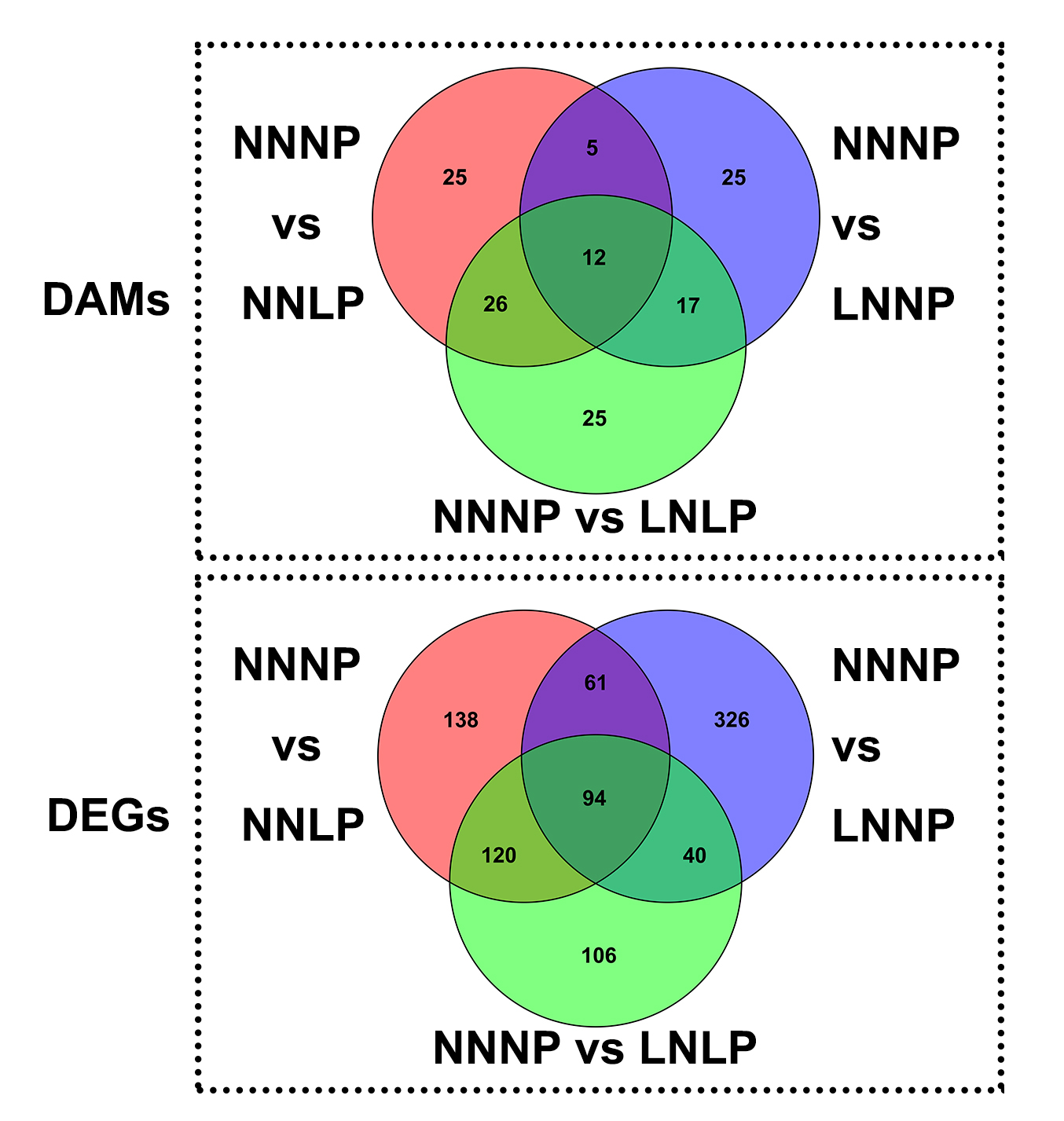

Supplement: Supplementary Figure 3 — Venn diagrams showing the overlap among DAMs and DEGs in each of the different conditions of N and P supply screened based on conjoint analysis. [file Image_3.jpeg]

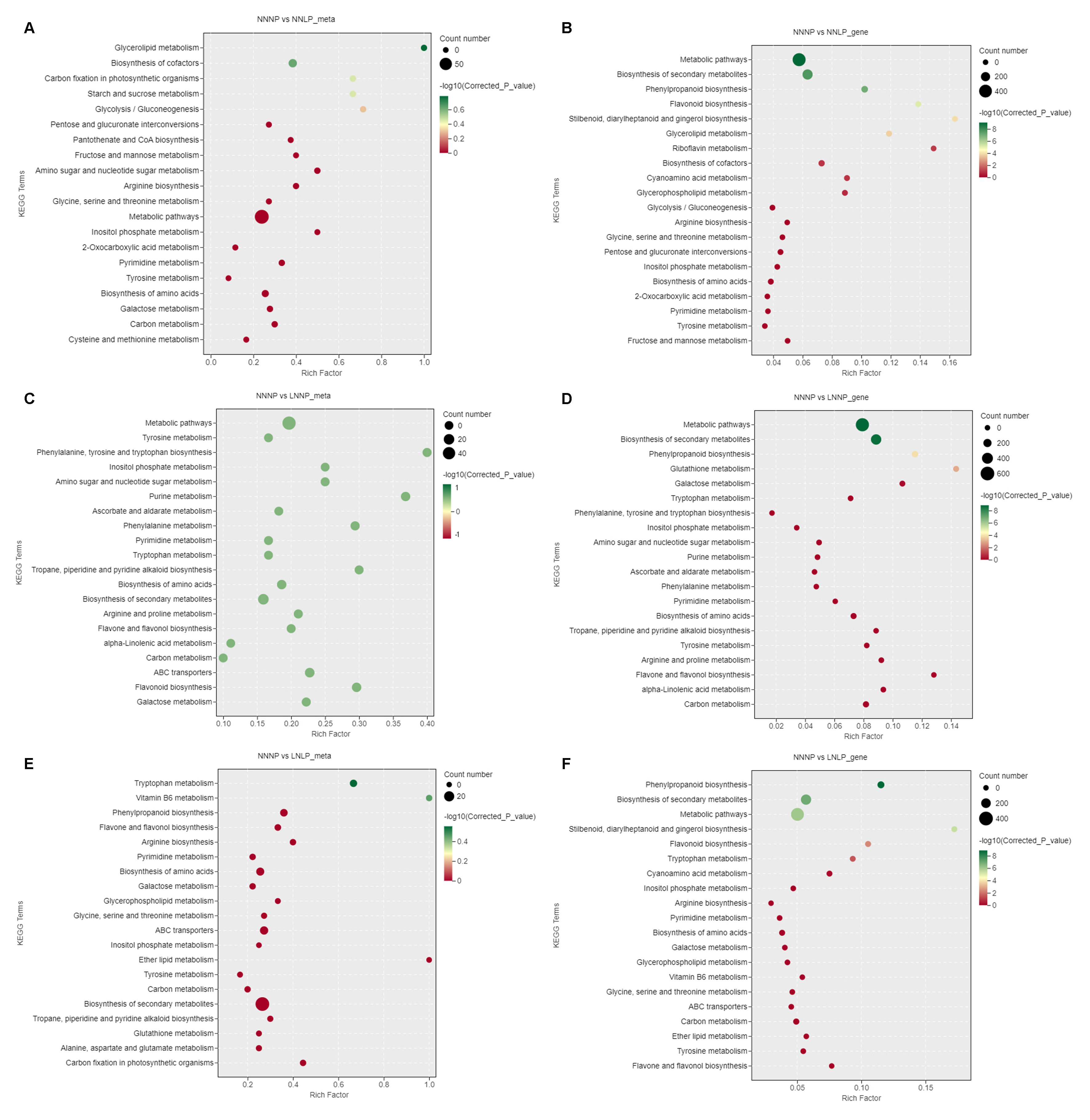

Supplement: Supplementary Figure 4 — Bubble diagram showing the divergence of the KEGG pathways contained DAMs and DEGs in response to N and/or P deficiency screened based on conjoint analysis. The horizontal coordinate represents the enrichment factor (Diff/Background) of the pathway in different histologies, and the vertical coordinate represents the name of the KEGG pathway; the red-yellow-green gradient represents the change in the significance of the enrichment from high to medium to low, and is indicated by -log10(P value); and the size of the bubble represents the number of DAMs or DEGs, the larger the number, the larger the point. [file Image_4.jpeg]

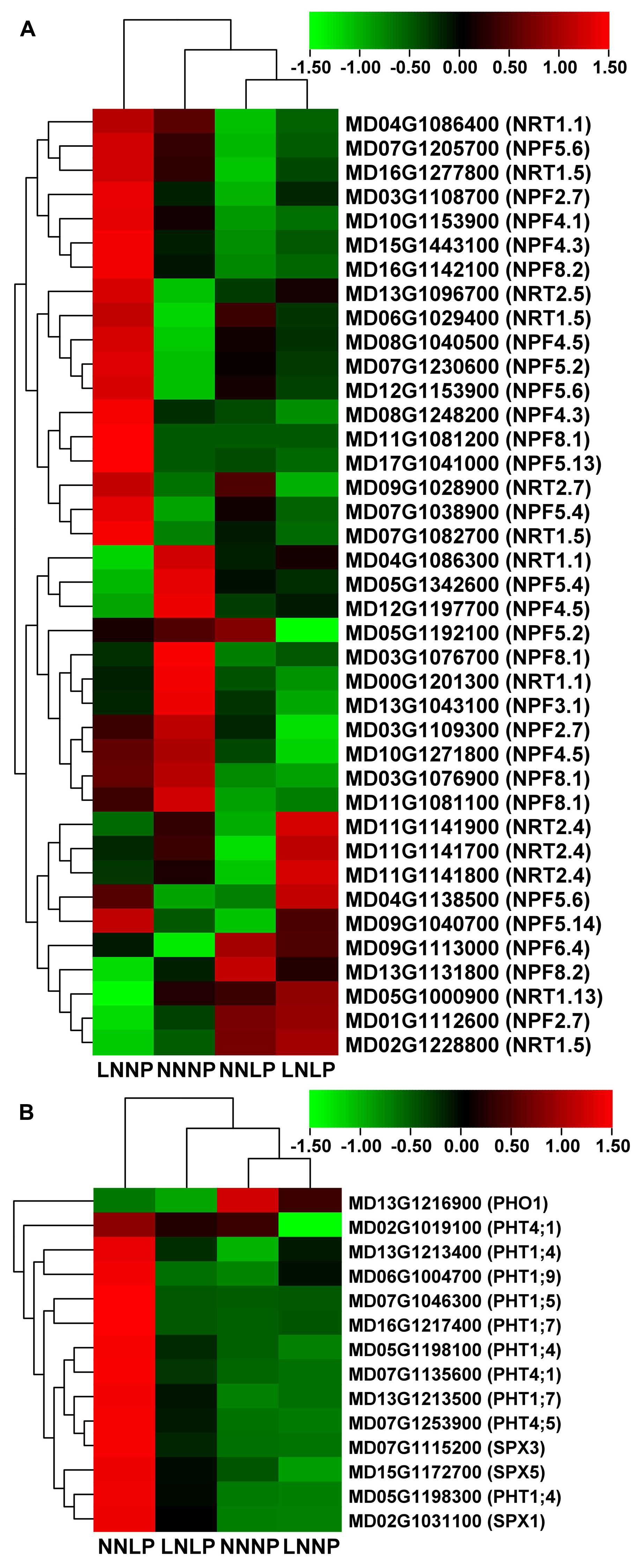

Supplement: Supplementary Figure 5 — Heatmap of DEGs involved in uptake and transport of N (A) and P (B) under different N and P supply conditions. [file Image_5.jpeg]

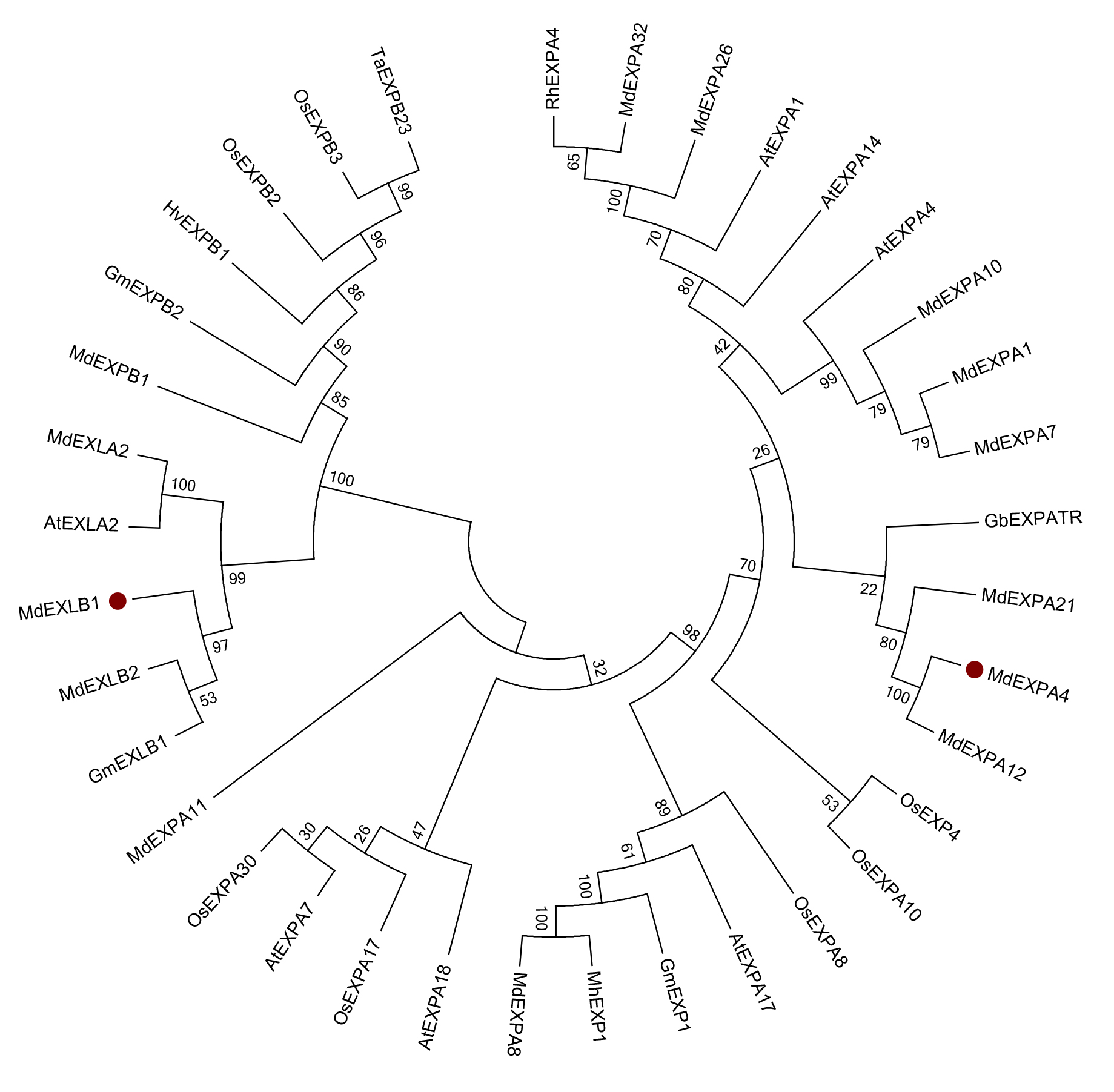

Supplement: Supplementary Figure 6 — Phylogenetic relationship of expansin genes involved in root development and growth from Arabidopsis thaliana, Oryza sativa, Hordeum vulgare, Glycine max, Rosa rugosa, Triticum aestivum, Gossypium arboretum, and Malus hupehensis. [file Image_6.jpeg]

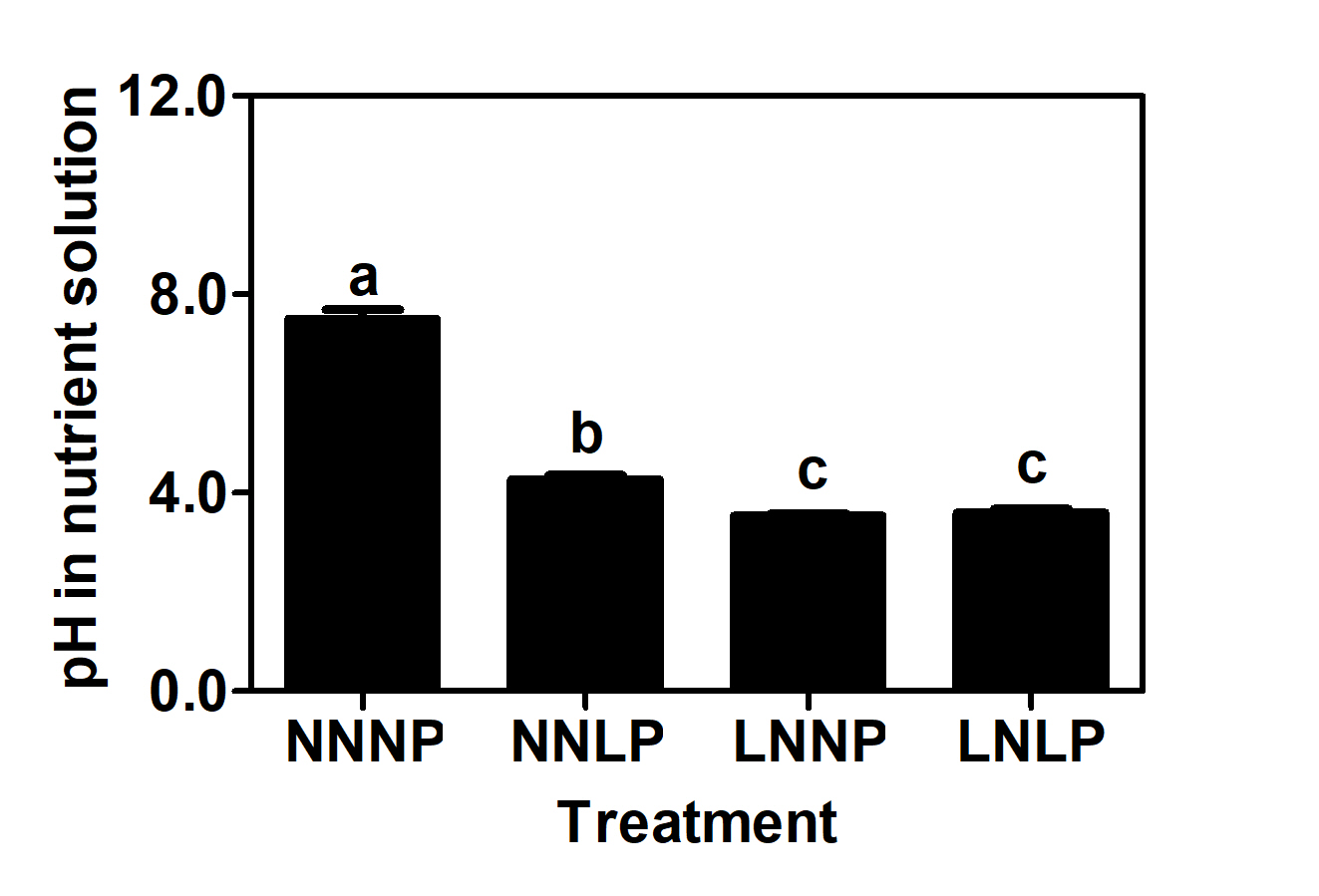

Supplement: Supplementary Figure 7 — Differences in pH of nutrient solution with different N and P concentrations on Day 60. [file Image_7.jpg]

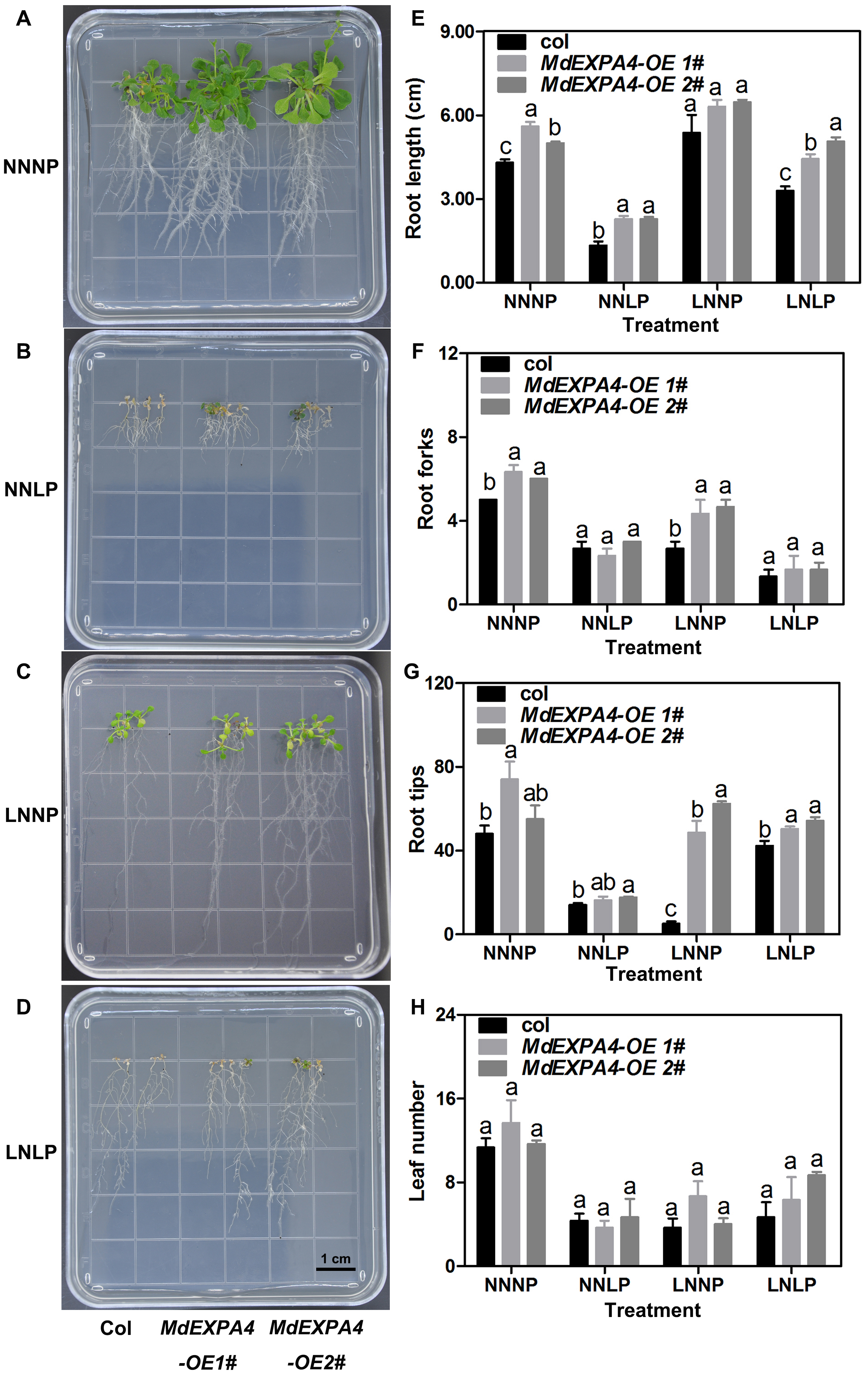

Supplement: Supplementary Figure 8 — Phenotypes of MdEXPA4 overexpressing lines subjected to NNNP, NNLP, LNNP, and LNLP conditions based on MS culture medium for two weeks were showed in A–D, respectively. Bar=1 cm. E–H, the root parameters of A. thaliana under different N and P supply conditions. [file Image_8.jpeg]

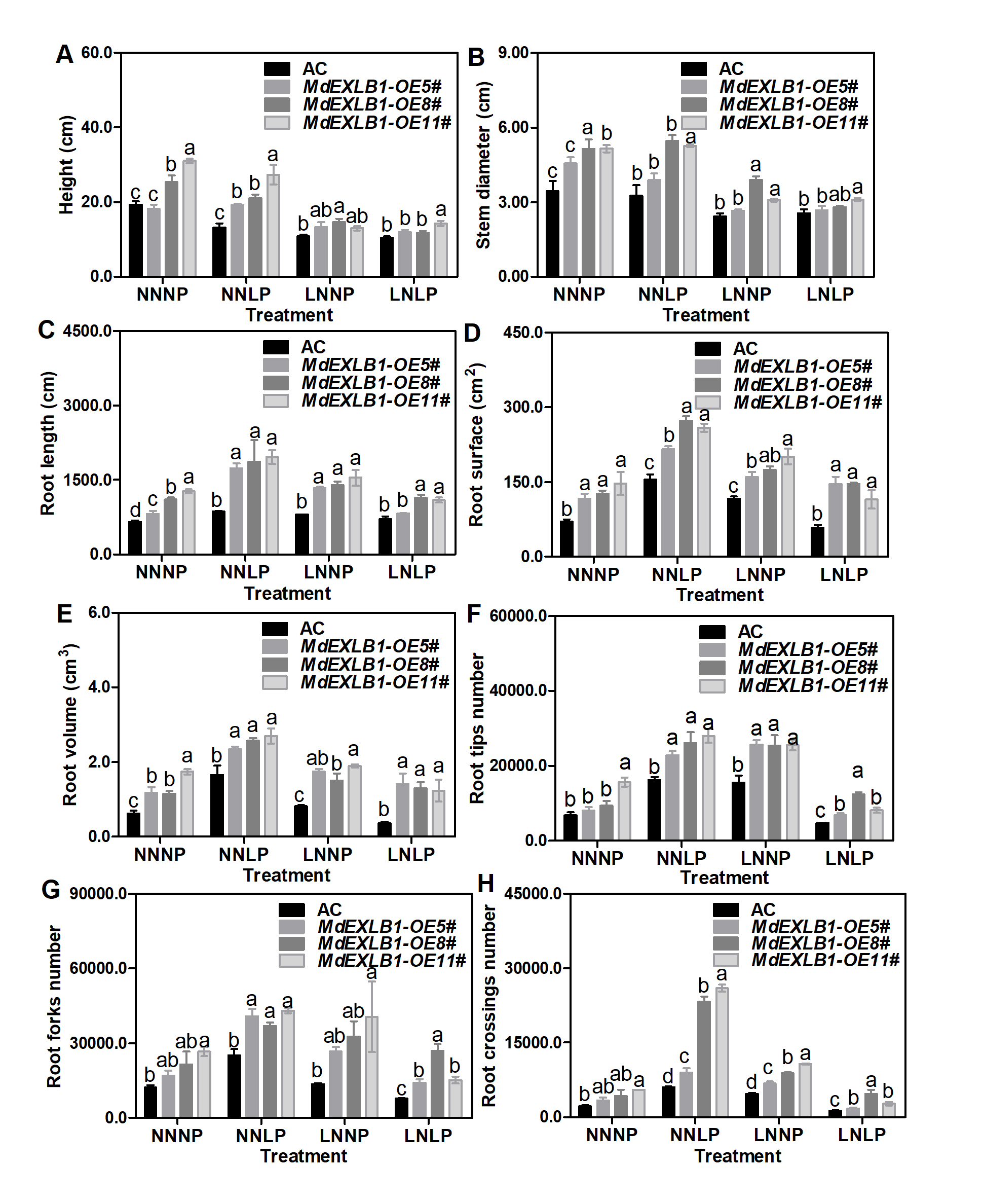

Supplement: Supplementary Figure 9 — Differences in the growth and root parameters of wild-type and MdEXLB1 overexpressing lines under different N and P supply conditions. [file Image_9.jpg]

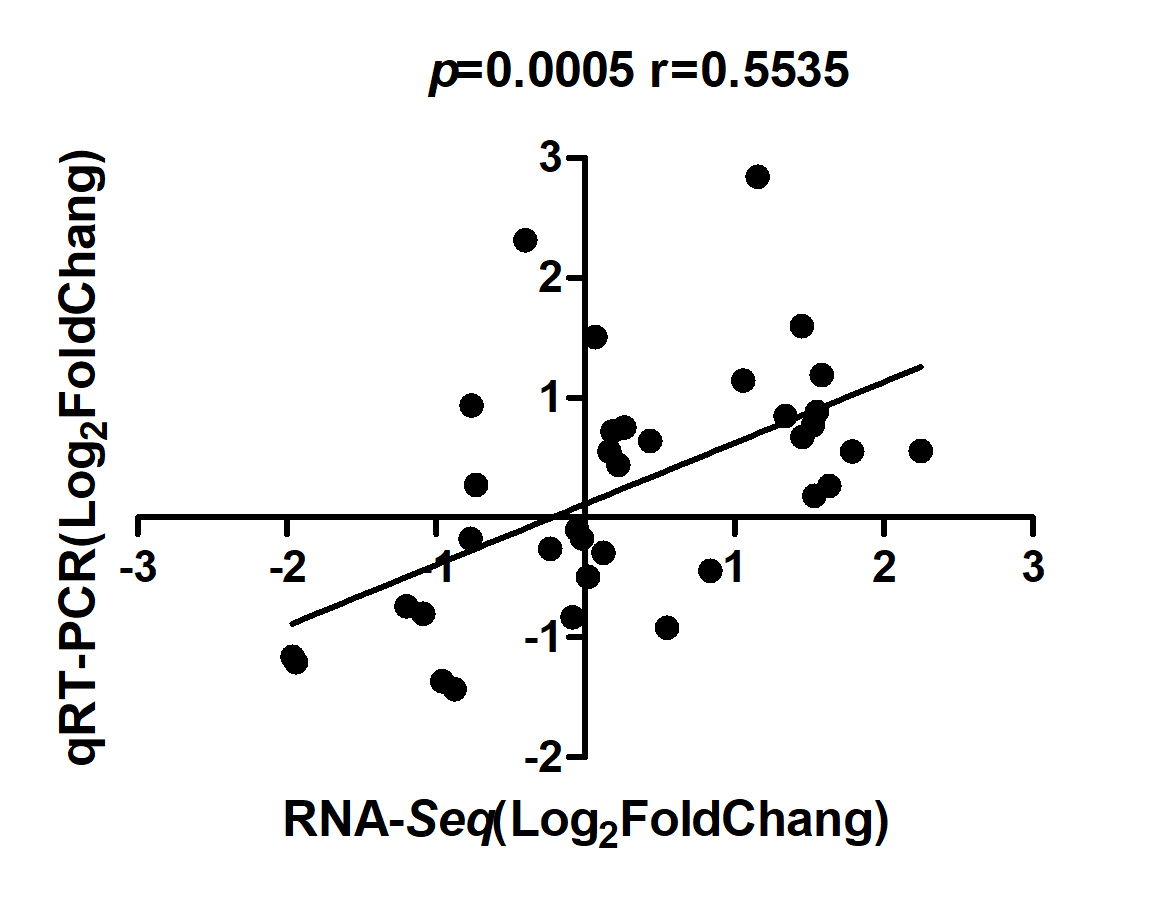

Supplement: Supplementary Figure 10 — Correlation between RNA-Seq and qRT-PCR data. Each RNA-Seq expression data was plotted against qRT-PCR data and fitted into a linear regression. [file Image_10.jpg]
